# Exosomes: a double‐edged sword in cancer immunotherapy

**DOI:** 10.1002/mco2.70095

**Published:** 2025-02-17

**Authors:** Jiayi Chen, Siyuan Hu, Jiayi Liu, Hao Jiang, Simiao Wang, Zhaogang Yang

**Affiliations:** ^1^ School of Life Sciences Jilin University Changchun China

**Keywords:** exosome, immune stimulatory, immune suppressive, tumor

## Abstract

Over the past few decades, immunotherapy has emerged as a powerful strategy to overcome the limitations of conventional cancer treatments. The use of extracellular vesicles, particularly exosomes, which carry cargoes capable of modulating the immune response, has been extensively explored as a potential therapeutic approach in cancer immunotherapy. Exosomes can deliver their cargo to target cells, thereby influencing their phenotype and immunomodulatory functions. They exhibit either immunosuppressive or immune‐activating characteristics, depending on their internal contents. These exosomes originate from diverse cell sources, and their internal contents can vary, suggesting that there may be a delicate balance between immune suppression and stimulation when utilizing them for immunotherapy. Therefore, a thorough understanding of the molecular mechanisms underlying the role of exosomes in cancer progression is essential. This review focuses on the molecular mechanisms driving exosome function and their impact on the tumor microenvironment (TME), highlighting the intricate balance between immune suppression and activation that must be navigated in exosome‐based therapies. Additionally, it underscores the challenges and ongoing efforts to optimize exosome‐based immunotherapies, thereby making a significant contribution to the advancement of cancer immunotherapy research.

## Graphical abstract

Immunotherapy has emerged as a potent strategy for overcoming the limitations associated with conventional cancer therapies. The use of extracellular vesicles, particularly exosomes which carry cargoes capable of regulating the immune response, has been widely discussed as a potentially therapeutic approach in cancer immunotherapy. Exosomes can transport their cargo to target cells, thereby influencing their phenotype and immunomodulatory functions. They exhibit either immunosuppressive or immune‐activating characteristics depending on their internal contents. These exosomes originate from diverse cell sources and their internal contents differ, providing an initial indication that there may be a fine line between immune suppression and stimulation when using them for immunotherapy. This review aims to delineate the roles of exosomes of various origins in cancer development and summarize their mechanisms of action.

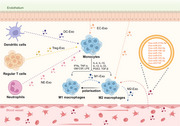

## 1 INTRODUCTION

Globally, cancer is a leading cause of death, responsible for approximately 10 million deaths in 2020.^1^ It is a progressive and complex process characterized by the accumulation of multilevel reactions and gene mutations. During this process, cancer cells adopt an immunosuppressed functional state and infiltrate normal tissues.^2^, ^3^ In recent years, there has been growing attention on the tumor microenvironment (TME), a highly structured ecosystem in which cancer cells are surrounded by various nonmalignant cell types embedded in an altered, vascularized extracellular matrix (ECM).^4^ Its homeostasis is affected by interactions across all cellular compartments, including malignant, endothelial, stromal, and immune cells.^5^, ^6^ Mounting evidence suggests that tumor growth and development depend not only on interaction between tumor cells but also on communication between tumor cells and other stromal cells.^7^, ^8^, ^9^ This intercellular communication forms the foundation of the tumor metastasis microenvironment, indicating that tumor invasion and metastasis rely on bidirectional communication between tumor cells and “nontumor” cells within the microenvironment.^10^ Furthermore, these complex cellular interactions often involve extracellular metabolites that acts as communication signals between different cells,^11^ and the modulation of these signaling networks may influence tumor progression. Studying the TME uncovers the mechanisms of immunosuppression and offers new insights for cancer immunotherapy.^12^, ^13^, ^14^ This field holds great promise as a novel therapeutic approach in the fight against cancer.^15^ Advances in cancer immunotherapy include immune checkpoint inhibitors, cell therapies (such as CAR T‐cell therapy), tumor vaccines, and immunomodulators.^16^, ^17^

Recently, significant progress has been made in the research of exosomes in cancer biology and immunotherapy. By secreting exosomes, tumor cells shape the local microenvironment,^18^ leading to immunosuppression and inhibiting the function of effector T cells and natural killer (NK) cells, thereby evading host immune surveillance. Furthermore, exosomes significantly influence the regulation of immune responses by modulating the activity and function of immune cells.^19^ As a result, their potential applications in cancer immunotherapy are evident. In this review article, we systematically summarize how exosomes derived from different cell types exert either immunosuppressive or immunostimulatory effects and discuss their applications in cancer therapy.

## 2 CHARACTERISTICS OF EXOSOMES

**FIGURE 1:**
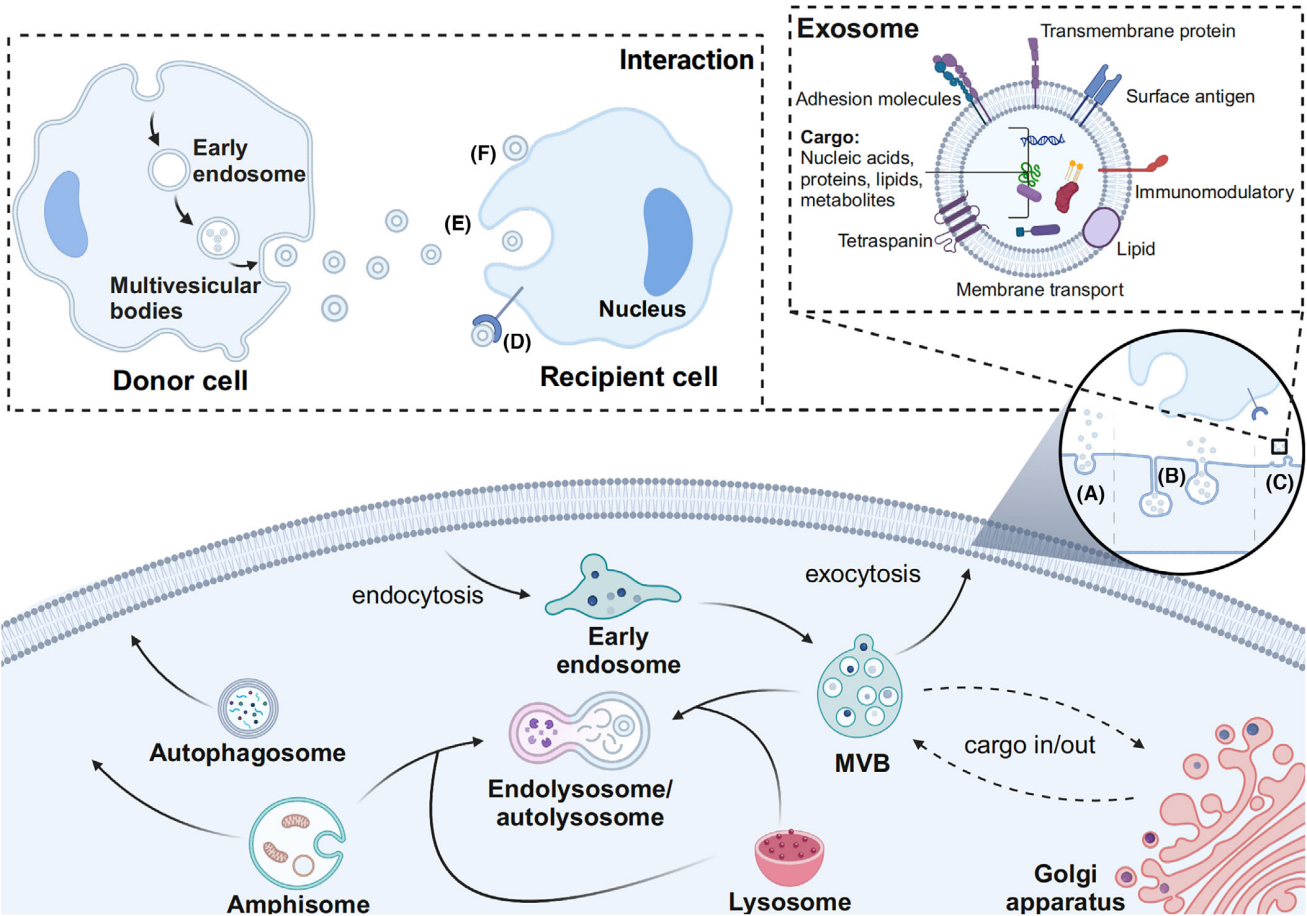
Exosome biogenesis: (a) exosome biogenesis mainly occurs through the endogenous multivesicular body pathway; (b) exosomes can also bud within intracellular compartments connected to the plasma membrane; (c) exosomes may directly bud from the plasma membrane. Exosomes interact with specific receptor cells through (d) receptor–ligand binding, (e) phagocytosis of cellular exosomes by the receptor cell, and (f) direct fusion of exosomes with receptor cell membranes. MVB, multivesicular body (created in BioRender.com).

Exosomes, with a size range of approximately 40–150 nm,^20^, ^21^ originate from endosomes and are extracellular vesicles produced by most, if not all, cells. They carry nucleic acids, proteins, lipids, and metabolites, either on their surface or within their internal structure.^22^, ^23^, ^24^, ^25^ Exosome biogenesis occurs through three primary modes^26^ (Figure 1). Traditionally, the most common and classical mode is the endogenous multivesicular body pathway.^20^ An alternative mode involves vesicles budding within intracellular compartments connected to the plasma membrane and eventually releasing exosomes.^27^ A third, more controversial mode suggests that vesicles may directly bud from the plasma membrane, leading to the immediate release of exosomes.^28^ However, whether this mechanism applies to all exosomes and its biological significance remain subjects for further investigation. Once released, exosomes play a significant role in human health and disease, including processes such as development, immunity, tissue homeostasis, and cancer.^29^, ^30^, ^31^ Since their discovery, exosomes were initially considered cellular debris, a perception that discouraged researchers from studying them in depth. However, an increasing number of studies have revealed that exosomes can act as signaling nanocarriers, delivering bioactive molecules (such as nucleic acids, proteins, and lipids) to specific recipient cells, thereby mediating intercellular communication under both physiological and pathological conditions and influencing various aspects of cell biology. For example, proteins expressed on the surface of exosomes not only enable exosomes to interact with membrane proteins on specific recipient cells through ligand–receptor mechanisms for subsequent cell‐to‐cell communication^32^ (Figure 1) but also prolong their blood circulation and promote tissue‐directed delivery.^33^ Moreover, exosomes are involved in activating the immune system, communicating with the nervous system, and regulating cell proliferation, homeostasis, and maturation.^34^, ^35^, ^36^, ^37^

In addition to their crucial role in regulating normal physiological processes, exosomes are also actively involved in pathological carcinogenesis.^38^, ^39^ In recent years, they have been extensively studied in the context of cancer development. During cancer progression, exosomes carry contents such as proteins, lipids, and nucleic acids, which are released outside the cell to participate in intercellular communication, immune responses, angiogenesis, and tumor cell growth.^40^ The function of exosomes, which can modulate cells in cancer by triggering either protumor or antitumor immune responses,^41^ may vary depending on the type of cancer, its stage of development, or the conditions under which they are obtained.^42^, ^43^ Furthermore, they contain a series of membrane‐associated high‐order oligomeric protein complexes and are classified into different subgroups based on their diverse molecular cargo, resulting in a high degree of heterogeneity.^26^ The heterogeneity of exosomes can result in distinct biological functions of exosomes depending on their source, thereby contributing to their varied properties. For instance, tumor‐derived exosomes (TEXs) can induce immune escape, thereby promoting tumor progress.^44^, ^45^ Studies have revealed that TEXs contribute to tumor progression and metastasis by influencing apoptosis, cellular immunity, angiogenesis, cellular metabolism, invasion, and metastasis. As a result, they remodel the TME by altering the phenotype of the stromal cells surrounding the tumor.^46^, ^47^ In contrast, TEXs exposed to radiotherapy, UV, or acid–base stimulation exhibit immunostimulatory effects.^48^, ^49^, ^50^ This suggests that exosomes can perform functions similar to those of their parental cells, with the cargo inside exosomes reflecting the characteristics of these cells.^51^

Recently, exosomes have attracted considerable attention because of their potential as natural carriers for drug delivery. The rapid development of nanotechnology, along with the growing interdependencies among basic biology, chemistry, and pharmacology, has enabled the encapsulation of functional molecular cargo (such as nucleic acids, proteins, etc.) within exosomes. These cargo molecules can be targeted to recipient cells with high specificity and bioavailability. In general, functional molecules can be loaded into exosomes using methods such as electroporation, freeze–thaw cycles, or sonication,^52^, ^53^ or they can be loaded during exosome biogenesis.^54^ Additionally, exosomes have endogenous functions, including excellent target homing specificity and immune surveillance, which endow them with intrinsic biophysical and biochemical properties for the treatment of a wide range of diseases. Therefore, exosomes have been evaluated as potential therapeutic agents in various disease models. However, as mentioned earlier, exosomes are heterogeneous, and their biological properties differ depending on their source. A systematic understanding of the biological characteristics of exosomes from different origins is essential for harnessing their advantages in the development of drug delivery systems. Overall, depending on their origins, exosomes act as double‐edged swords in cancer development, exhibiting either immunosuppressive or immune‐activating functions.

## 3 THE IMMUNE SUPPRESSIVE EFFECTS OF EXOSOMES

**FIGURE 2:**
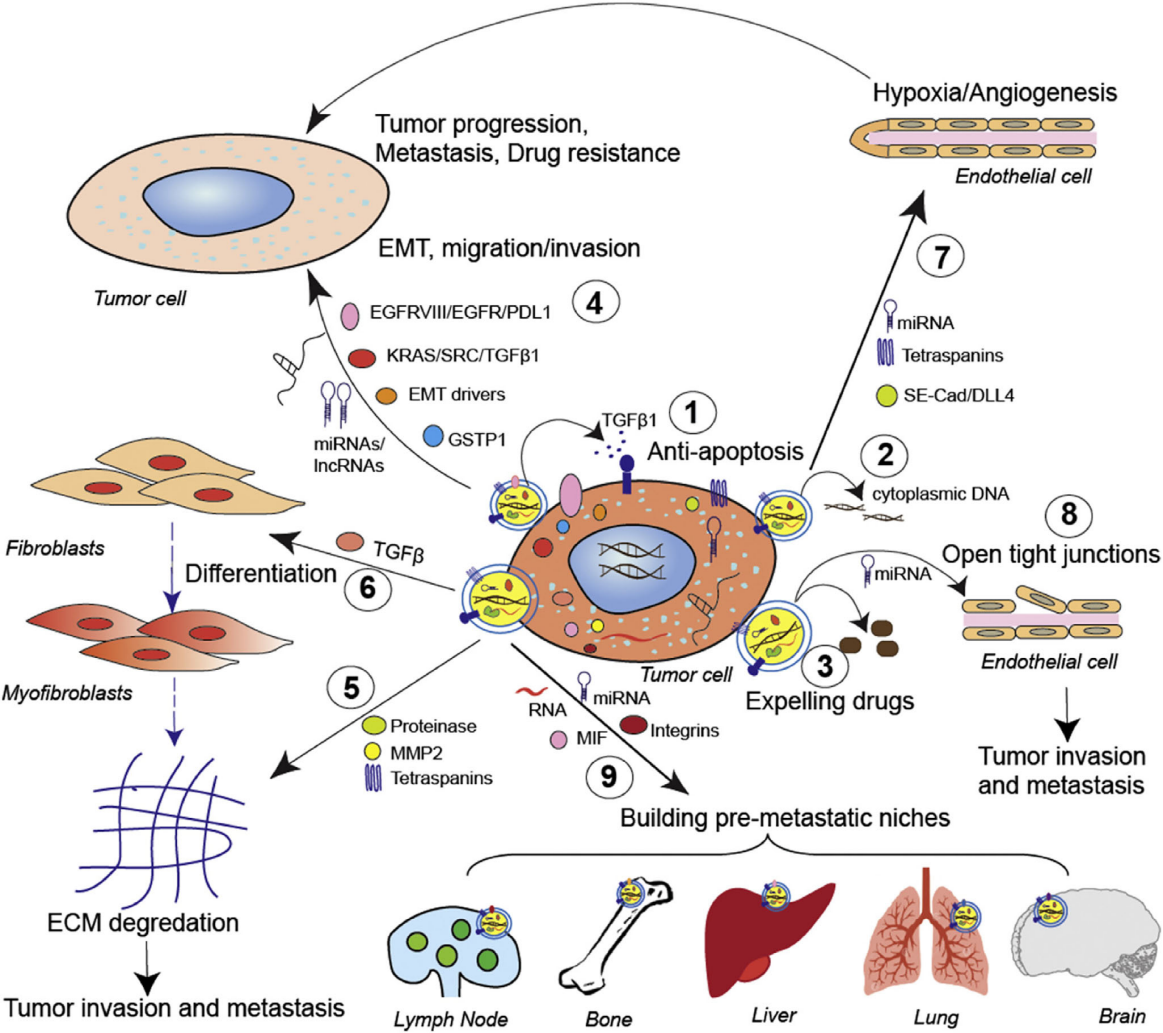
The role of TEXs in tumor progression is as follows: (1) TEXs inhibit apoptosis in tumor cells by secreting transforming growth factor‐β (TGF‐β)1, and (2) they secrete cytoplasmic DNA for cellular homeostasis, (3) and also can release cytotoxic drugs that lead to drug resistance in tumor cells. (4) TEXs transfer their cargo to other tumor cells, promoting epithelial–mesenchymal transition (EMT), migration, and invasion. TEXs can (5) reprogram the ECM or (6) induce fibroblasts to differentiate into myofibroblasts by secreting TGF‐β, which further induces ECM degradation; they can also (7) enhance endothelial cells (ECs) proliferation and angiogenesis, and (8) turn on tight junctions in ECs via exosomal microRNA(miR)s, leading to tumor invasion and metastasis. (9) TEXs carrying specific integrins, mRNAs, or miRs can promote the establishment of premetastatic niches (reprinted with permission from Ref.^62^).

TEXs have been extensively studied in various types of cancer, including breast cancer (BC), lung cancer, and melanoma. Compared with normal cells, tumor cells typically produce more exosomes, and TEXs have a pronounced ability to modulate both local and distant microenvironments. TEXs can promote tumor cell survival by reducing proapoptotic signaling in donor cells or enhancing antiapoptotic signaling in recipient cells. Recent evidence has revealed that TEXs carry bioactive molecules that interact with various cell types in the TME, modulating cancer‐associated processes such as immune suppression and cancer metastasis^55^, ^56^ (Figure 2). For example, TEXs may contain molecules such as PD‐L1 and transforming growth factor beta (TGF‐β), which are capable of mediating immunosuppression.^57^, ^58^ By delivering these bioactive molecules to recipient cells, TEXs can alter the cellular physiology of both surrounding and distant normal cells, condition premetastatic sites in distant organs,^59^ disrupt endothelial barrier integrity, and induce angiogenesis.^60^ Therefore, they facilitate metastasis and growth of cancer cells. Moreover, by providing substrates that promote cell adhesion and increase cell motility through the binding of integrins on TEXs to ECM components such as fibronectin, TEXs can also facilitate directed cell movement.^61^ In addition to TEXs, studies have indicated that exosomes derived from stromal and infiltrating immune cells, including bone marrow‐derived cells, regulatory T cells (Tregs), and M2 macrophages, can suppress the response of solid tumors to immunotherapy (Table S1). The role of these exosomes in the TME is complex and dynamic, forming a mutually reinforcing network that drives tumor growth and metastasis. At the molecular level, exosomes suppress immunity by preparing a premetastatic niche, promoting angiogenesis, remodeling stromal cells, reprogramming metabolism, and facilitating immune evasion. These processes are interconnected, creating a TME that supports tumor growth, invasion, and metastasis while evading immune surveillance. Understanding these processes is crucial for developing therapies that can block exosome‐mediated immune suppression, potentially improving the outcomes of immunotherapy.

### 3.1 Promoting angiogenesis

**FIGURE 3:**
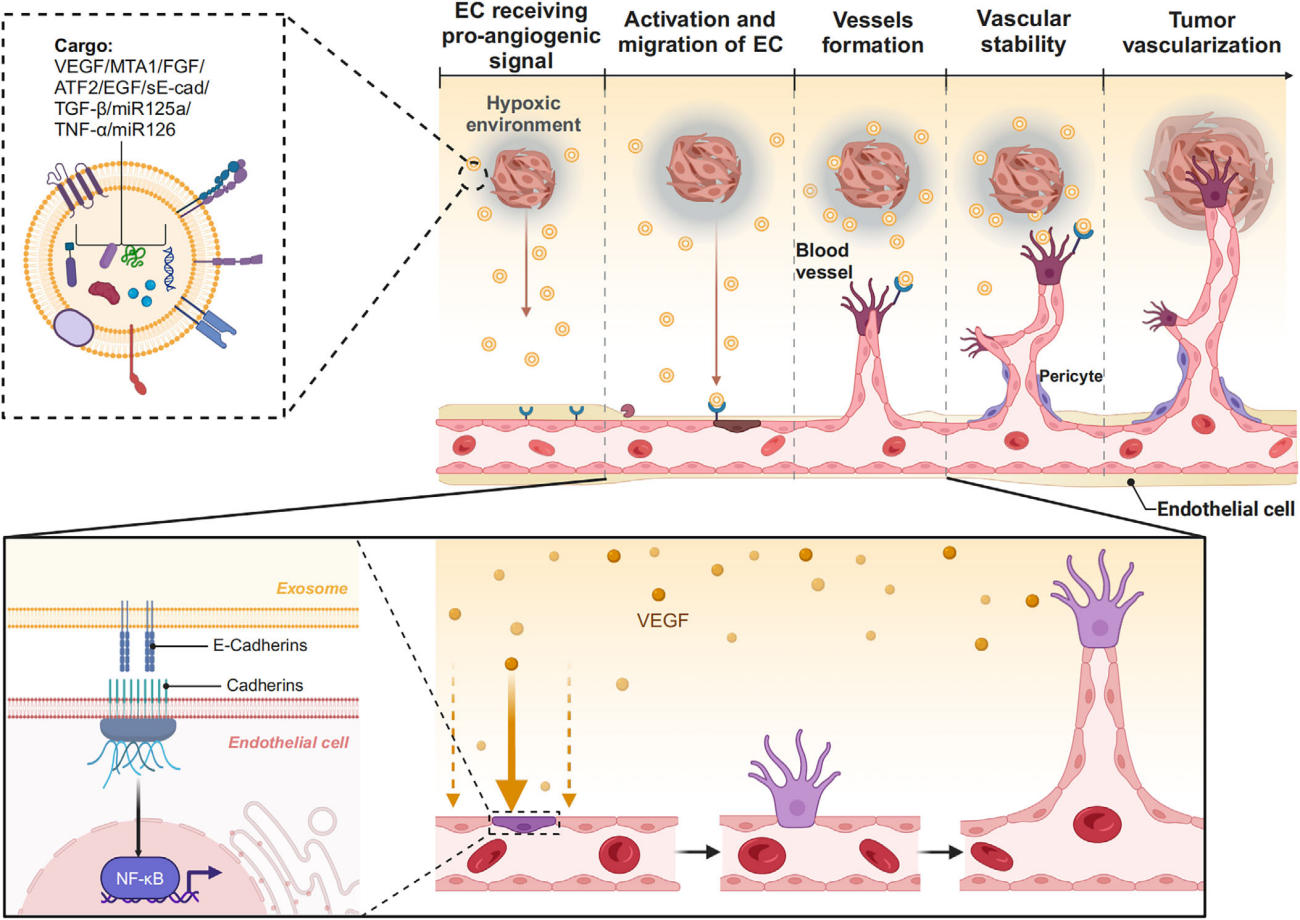
The process of angiogenesis is promoted by exosomes. TEXs carry various proangiogenic factors that function at different stages. EC receives provascular signals (VEGF signaling) from TEXs and is activated. The activation then stimulates EC migration and enhances overall vascular permeability, leading to the formation of new blood vessels. Eventually, after vascular stabilization, the tumor becomes vascularized (created in BioRender.com).

Angiogenesis, a hallmark of cancer, is essential for tumor progression. It is typically triggered by low oxygen levels within the tissue, which lead to the expression of multiple growth factors by cancer cells and stromal cells, such as fibroblasts and macrophages. These stromal cells are recruited to the tumor site through the action of hypoxia‐inducible factors. In cancer progression, pathological angiogenesis is driven by the overexpression of proangiogenic factors, creating a local imbalance between pro‐ and antiangiogenic signals and facilitating the recruitment of a new vascular supply.^63^ Angiogenesis within tumors is associated with the formation of an immunosuppressive microenvironment,^64^ which facilitates the recruitment of immunosuppressive cells,^65^ including Tregs and myeloid‐derived suppressor cells (MDSCs).^66^ This process results in the secretion of immunosuppressive factors that inhibit effector T cell activity. Angiogenesis involves five main steps,^67^ and some TEXs contain various proangiogenic factors that play a regulatory role at different stages of cancer angiogenesis (Figure 3). First, endothelial cells (ECs) receive proangiogenic signals from factors such as vascular endothelial growth factor (VEGF), platelet‐derived growth factor (PDGF), placental growth factor, epidermal growth factor, fibroblast growth factor, TGF‐β1, and tumor necrosis factor alpha (TNF‐α).^68^, ^69^ Because exosomes carry these factors, they play an important role in EC receiving provascular signals. For example, VEGF present in exosomes can promote angiogenesis through VEGF signaling.^70^ Second, ECs are activated and migrate, and exosomes are involved in this process. For example, TEXs derived from the ascites of patients with ovarian cancer contain E‐cadherin, which acts as a potent activator of angiogenesis in a VEGF‐independent manner.^71^ At the molecular level, the binding of E‐cadherin‐positive exosomes to calmodulin on ECs drives a signaling cascade that ultimately activates β‐linked protein and NF‐κB. Third, this activation then stimulates ECs migration and enhances overall vascular permeability, leading to the formation of new blood vessels.^71^ A recent study showed that exosomes from ovarian cancer enhance the viability of human umbilical vein ECs (HUVECs).^72^ Proteomic analysis further revealed that activating transcription factor 2 and metastasis‐associated protein 1, present in ovarian cancer exosomes, are responsible for the upregulation of angiogenesis. Additionally, owing to its ability to regulate matrix metalloproteinase expression in stromal cells surrounding the tumor, CD147‐positive exosomes secreted by ovarian tumors can also promote blood vessel formation in HUVECs.^73^ Next, the maturation and stabilization of blood vessels are essential for the development of new vascular networks. During this process, the migration of pericytes is a key step, as they are closely associated with vascular ECs and line the exterior of blood vessels,^74^ aiding in the maturation and stabilization of neovascularization through their interactions with ECs. Eventually, after vascular stabilization, the tumor becomes vascularized. Exosomes containing PDGF–BB recruit pericytes to the newly formed blood vessels, further stabilizing them by secreting angiopoietin‐1.^75^, ^76^ Similarly, BC‐derived exosomes transmit oncogenes and proangiogenic signals to ECs, thereby promoting tumor vascularization.^77^ In addition to priming angiogenesis, the proangiogenic microenvironment increases vascular permeability to multiple cell types, including VEGFR1^+^ hematopoietic progenitors, immune cells, stromal cells, and tumor cells.

Overall, angiogenesis enables the premetastatic niche to meet the nutritional demands required for subsequent rapid metastatic growth.^78^ It supplies oxygen and nutrients to tumors while also creating pathways for tumor cell migration and dissemination. The stimulation of angiogenesis in adjacent tissues through the release of signaling molecules, such as exosomal VEGF, not only facilitates tumor growth but also provides vascular support for the establishment of premetastatic niches.

### 3.2 Preparing premetastatic niche

**FIGURE 4:**
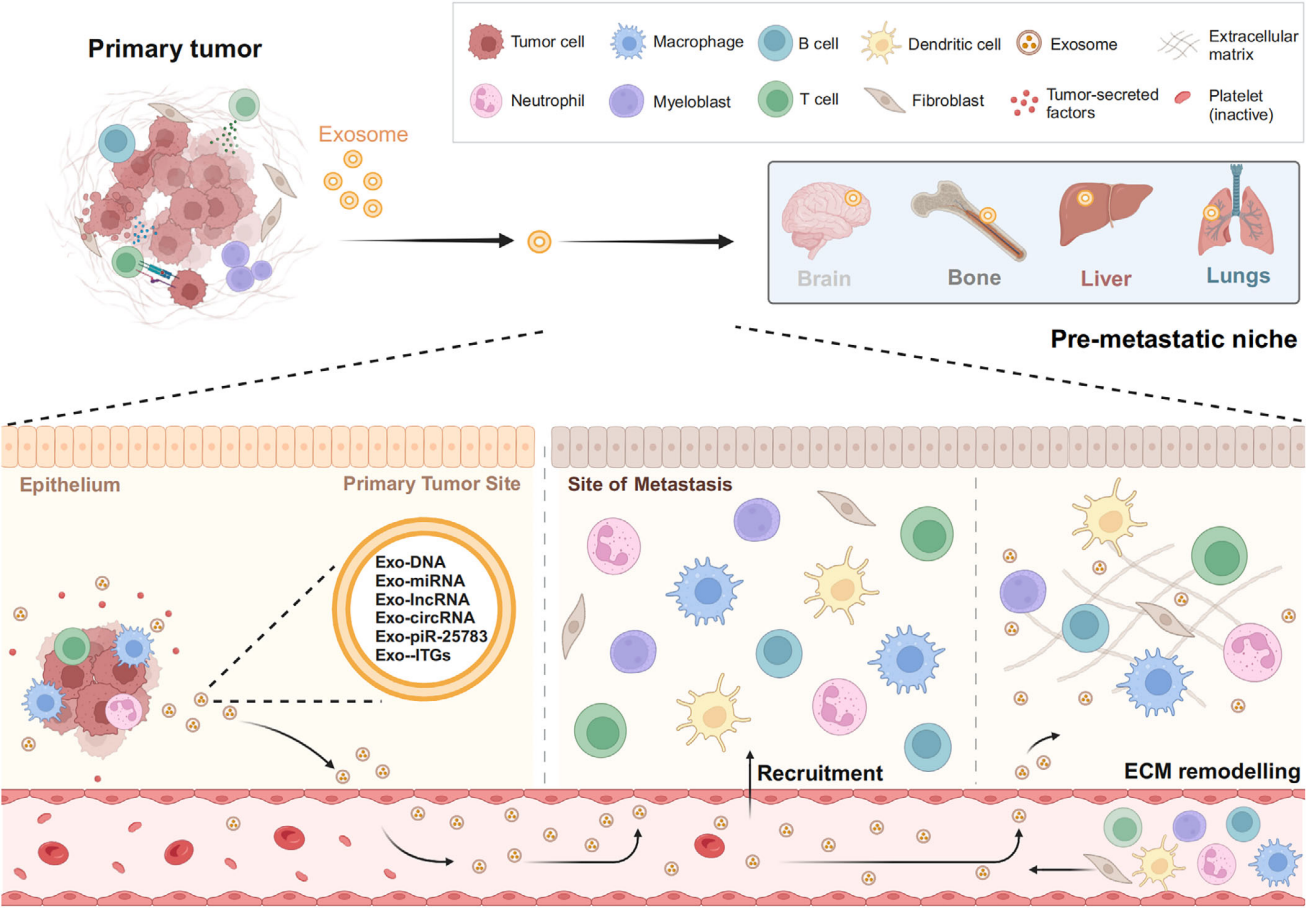
Effects of exosomes on tumor premetastatic niche formation. Exosomes secreted by tumor cells can specifically recruit immune cells, fibroblasts, and ECs into premetastatic niches, synergistically supporting tumor cell colonization and growth. Exosomes secreted by tumor cells affect the remodeling of ECM and create favorable conditions for the migration and colonization of tumor cells (created in BioRender.com).

Enabling the proliferation of cancer cells in the premetastatic niche, a crucial step in the metastatic process, is essential for protecting metastatic cells from apoptosis. This process may also attract immunosuppressive cells, leading to a reduced local immune response,^79^ which can impact the effectiveness of immunotherapy. Recent studies have demonstrated that TEXs contribute to premetastatic niche formation by remodeling of ECM and recruiting immune cells, fibroblasts, and ECs (Figure 4).^80^, ^81^ It was found that exosomal migration inhibitory factor (MIF) induces the release of TGF‐β by Kupffer cells, which in turn promotes fibronectin production by hepatic stellate cells. Fibronectin facilitates the arrest of macrophages and neutrophils in the liver, contributing to the formation of a premetastatic niche in pancreatic ductal adenocarcinoma.^59^ Therefore, knocking down or blocking MIF could serve as a potential strategy to prevent exosome‐induced metastasis in pancreatic ductal adenocarcinoma. Additionally, it has been found that communication between cancer cells and omental fibroblasts can be mediated by exosomal piR‐25783, which promotes tumor metastasis.^82^ Exosomal piR‐25783 released from ovarian cancer cells activates the TGF‐β/SMAD2/SMAD3 pathway in fibroblasts and promotes the differentiation of omental fibroblasts into myofibroblasts, leading to an increase in their proliferative, migratory, and invasive properties. This ultimately results in the formation of the premetastatic niche. Moreover, to investigate whether TEXs promote cancer cell colonization at specific organ sites, exosomes from breast and pancreatic cancer cells with liver or lung metastasis were selected for study.^83^ Notably, the results showed that TEXs were sufficient to redirect the metastasis of tumor cells with poor metastatic potential to specific organs. This result suggests that TEXs can create a favorable microenvironment that facilitates metastatic colonization in other tissues. A recent study further addressed the molecular mechanism by which exosomes direct the organ‐specific colonization of cancer cells. For example, exosomal integrins facilitate organ‐specific colonization by fusing with target cells in a tissue‐specific manner. This process initiates the formation of premetastatic niches.^83^ In summary, as discussed above, exosomes have the potential to facilitate the formation of premetastatic niches, thereby promoting cancer cell metastasis.

### 3.3 Remodeling stromal cells

Before metastasis, tumor cells alter the microenvironment of the distal organ through exosomes, inducing the remodeling of stromal cells. These changes enhance the supportive matrix and promote the establishment of an immunosuppressive environment,^84^ which favors tumor cell survival and metastasis. The condition of stromal cells significantly influences the recruitment and polarization of immune cells. Altered stromal cells may attract tumor‐promoting immune populations,^85^ such as M2 macrophages, while simultaneously hindering the infiltration of antitumor immune effectors, including CD8^+^ T cells and dendritic cells (DCs). Consequently, stromal cell remodeling can profoundly impact the effectiveness of immunotherapy.^86^ In addition to promoting angiogenesis and preparing premetastatic niches, exosomes are also actively involved in the remodeling of stromal cells, including fibroblasts, mesenchymal stem cells (MSCs), adipocytes, ECs, and pericytes.^87^ In particular, resting fibroblasts are activated to form cancer‐associated fibroblasts (CAFs), which are the most abundant cell type in the tumor stroma during tumor formation^88^ This activation is typically characterized by their ability to synthesize and remodel the ECM, alter their secretion profile, proliferate, and express specific markers, such as alpha‐smooth muscle actin.^89^ CAFs have been reported to contribute to cancer progression by interacting with tumor‐infiltrating immune cells and other immune components within the TME. They secrete various cytokines, growth factors, chemokines, exosomes, and other effector molecules, thereby creating an immunosuppressive TME that enables cancer cells to evade immune surveillance.^90^ Because exosomes possess some functions of their parental cells, those derived from CAFs also exhibit immunosuppressive effects similar to those of CAFs. Studies have shown that treatment with CAF‐derived exosomes increases the expression of miR‐92, which in turn promotes the migration and invasion of BC cells.^91^ Moreover, exosomes from other sources can influence the phenotype of fibroblasts and facilitate their conversion into CAFs. Recent studies have demonstrated that TEXs can regulate fibroblast activation into CAFs, thereby promoting tumor progression and metastasis.^92^, ^93^ miR‐1247‐3p from TEXs converts fibroblasts into CAFs by downregulating B4GALT3, thereby activating the β1‐integrin–NF‐κB signaling pathway and promoting lung metastasis of hepatocellular carcinoma.^94^ Moreover, hepatocellular carcinoma‐derived exosomes containing miR‐21 can also convert hepatic stellate cells into CAFs by downregulating PTEN and activating the PDK1/AKT signaling pathway, thereby promoting angiogenesis.^95^ Similarly, exosomes from cancer cells can induce adipose stromal cells to differentiate into tumor‐supporting myofibroblasts with tumor‐invasive properties.^96^ Furthermore, studies have shown that ovarian‐derived exosomes reprogram stromal cells into a tumor‐promoting stromal niche, facilitating metastatic colonization. A molecular mechanism study revealed that exosomal miR‐141 secreted by ovarian cancer cells mediates tumor–stroma interactions, leading to the formation of a tumor‐promoting stromal niche through activation of the YAP1/GROα/CXCRs signaling cascade.^97^ GROα, which promotes the proliferation and aggression of ovarian cancer cells, was drastically upregulated in primary stromal cells from stromal‐specific Yap1 conditional knockout mice. Exosomal miR‐141 targeted YAP1, leading to upregulation of GROα levels in stromal fibroblasts. Therefore, inhibiting exosomal miR‐141 secretion or blocking GROα receptors may slow down tumor progression and dissemination, offering a potential therapeutic approach to prevent ovarian cancer metastasis.

Moreover, exosomes can remodel the tumor niche by promoting the differentiation of MSCs into fibroblasts. This concept suggests that tumor cells, through exosome secretion, can “educate” MSCs to adopt a cancer‐initiating cell phenotype in the microenvironment.^98^ By interacting with surrounding stromal cells, exosomes can alter the properties and functions of these cells, creating a microenvironment more conducive to cancer progression. These alterations may enable cancer cells to invade surrounding tissues more effectively, evade immune detection, and develop resistance to treatment. Consequently, understanding how exosomes influence stromal cells and how to intervene in this process has become a crucial focus in cancer immunotherapy research.

### 3.4 Reprogramming of metabolism

Metabolic reprogramming is critical for cancer cells to adapt to their proliferation demands, the TME, and distant metastasis during tumor development.^99^ Tumors not only increase glucose uptake and glycolysis but also enhance glutamine consumption and catabolism, thereby providing essential carbon and amino nitrogen to support tumor growth.^100^, ^101^ Normally differentiated cells primarily rely on mitochondrial oxidative phosphorylation for cellular energy, whereas most tumor cells rely on aerobic glycolysis, a phenomenon known as the “Warburg effect.”^102^ Aerobic glycolysis is inefficient in ATP production but it offers several advantages to tumor cells, as its primary end product, lactate, can be taken up by tumor cells to replenish the tricarboxylic acid cycle.^103^ Furthermore, the accumulation of lactic acid impairs T cell functionality, hindering their ability to mount an effective response against tumor cells.^104^ Exosomes derived from BC cells are associated with cancer metastasis and can inhibit glucose consumption in fibroblasts by downregulating PKM2 through exosomal miR‐122. In addition, phosphorylated PKM2 can promote the secretion of exosomes, suggesting that aerobic glycolysis plays a crucial role in exosome secretion. More importantly, PKM2 is not only involved in exosome secretion but also plays a key role in remodeling the TME, further driving metabolic alterations in tumor stromal cells.^51^ In other words, metabolic reprogramming may also affect the stromal cell behavior, promoting their polarization toward protumor properties. By targeting metabolism signaling, exosomes have also been exploited as gene delivery vehicles to transfer short interfering RNA (siRNA) to cancer cells, effectively inhibiting glycolysis and reversing oxaliplatin resistance in drug‐resistant colorectal cancer cells.^105^

In addition to glucose and glutamine metabolism, lipid metabolism serves as another efficient pathway for cancer cells to generate energy.^106^ Specifically, cancer cells meet their proliferative demands by increasing the uptake of exogenous lipids and lipoproteins or promoting the synthesis of endogenous lipids. Recent studies have found that transcripts associated with lipogenesis and cholesterol synthesis pathways are upregulated in tumors, playing a critical role in the development and progression of various cancers.^107^, ^108^, ^109^ For example, ovarian cancer cells stimulate lipolysis in adipocytes to release lipids, which are then utilized as energy sources by the cancer cells, thus contributing to tumor progression. Similarly, exosomes are involved in reprogramming lipid metabolism in cancer cells. For instance, circ_0008285, a crucial mediator in the repression of low‐density lipoprotein receptor‐controlled lipid secretion, is downregulated in exosomes derived from the follicular fluid of polycystic ovarian syndrome. These exosomes are implicated in the disruption of ovarian lipid metabolism.^110^ Moreover, the pseudogene transmembrane protein 198B promotes enhanced lipid accumulation and fatty acid oxidation in macrophages through glioma‐derived exosomes, leading to immune escape of glioma cells.^111^ Additionally, elevated levels of HSPC111 in exosomes derived from colorectal cancer cells reprogram lipid metabolism in CAF by phosphorylating ATP‐citrate lyase. This, in turn, enhances the expression and secretion of CXCL5, which plays a crucial role in promoting cancer cell migration and metastasis.^112^

In short, exosomes play an important role in the TME by influencing metabolic reprogramming, which in turn affects the function of immune cells and the efficacy of immunotherapy. A deeper understanding of these mechanisms could provide valuable insights for developing more effective immunotherapeutic strategies.

### 3.5 Immune escape

**FIGURE 5:**
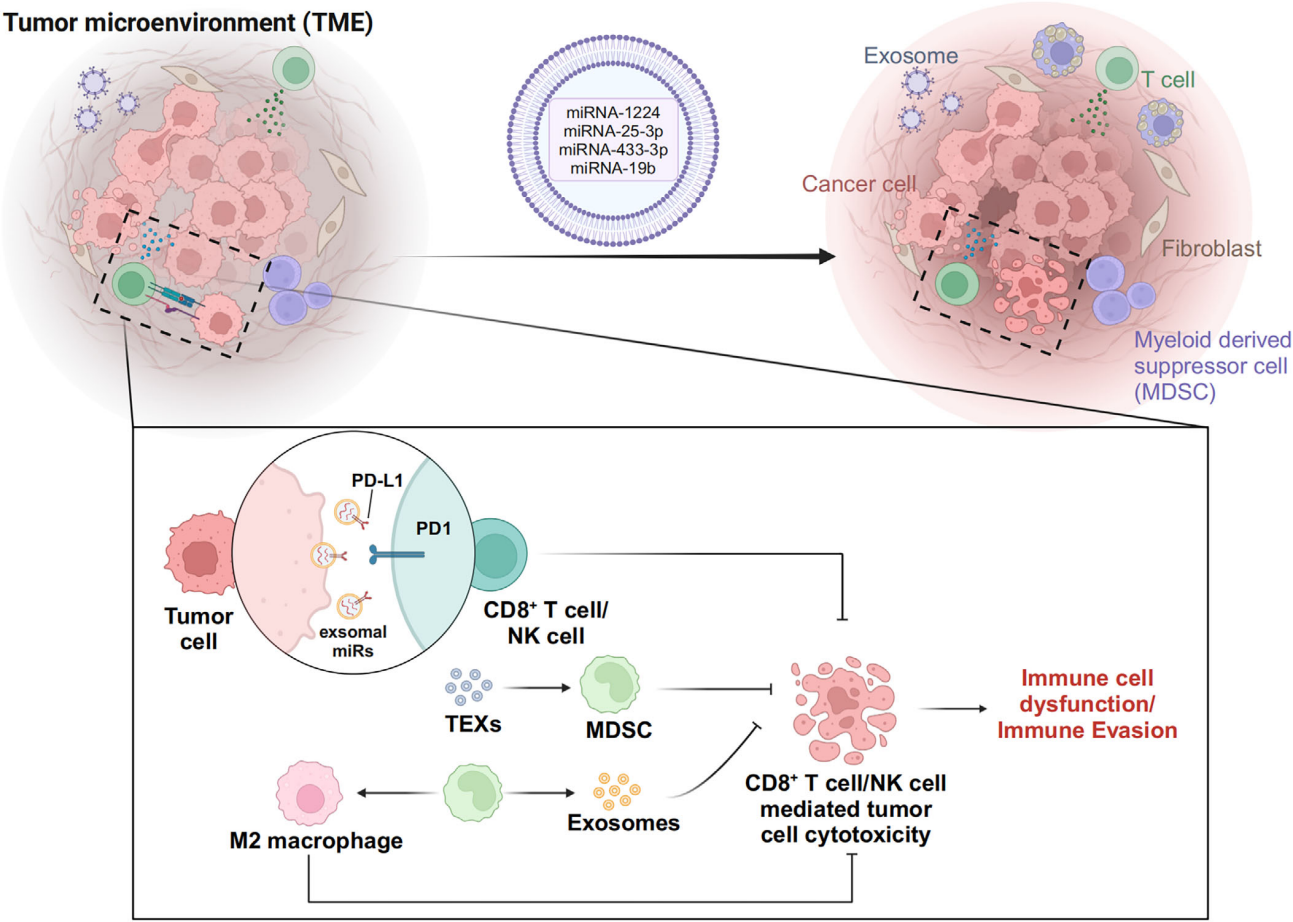
Schematic representation of the role of exosomes in promoting immune escape. PD‐L1 released from TEXs can transmit inhibitory signals to T cells expressing PD‐1, inhibit CD8^+^ T cell activity, and induce apoptosis of CD8^+^ T cells, thereby enabling tumor cell immune evasion. Exosomes derived from MDSCs can directly accelerate tumor cell proliferation and metastasis. MDSCs can suppress the immune response by inducing Tregs and promoting the differentiation of macrophages into the M2 phenotype (created in BioRender.com).

Evading immune detection and destruction is a critical hallmark and prerequisite for tumor cells to metastasize and sustain neoplastic progression. Under immune surveillance, cancer cells with high immunogenicity are preferentially eliminated by effector cells.^113^ However, cancer cells with low immunogenicity can evade immune clearance, allowing them to become the dominant subpopulation.^114^ The loss of immunogenicity is a crucial factor in cancer immune evasion. In addition, cancer cells can employ other strategies to evade the immune system, such as inducing regulatory immune cells, acquiring impaired antigen presentation capabilities, upregulating immune checkpoints, and creating an immunosuppressive microenvironment. Importantly, exosomes play an important role in facilitating these immune evasion mechanisms (Figure 5). Studies have shown that TEXs expressing PD‐L1 may serve as key mediators of tumor immune escape.^115^ PD‐L1 released from TEXs can transmit inhibitory signals to PD‐1‐expressing T cells, inhibit CD8^+^ T cell activity, and induce their apoptosis, thereby enabling immune evasion by tumor cells. In research conducted by Robert Blelloch, a syngeneic prostate cancer model, the TRAMP‐C2 model, was established to investigate whether exosomal PD‐L1 can promote tumor progression in vivo. The experimental study demonstrated that exosomal PD‐L1 can facilitate tumor progression, not only in the TRAMP‐C2 model but also in a colorectal model, where similar results were obtained. Besides, they identified exosomal PD‐L1 as a key regulator of tumor progression and its ability to inhibit T cell activation in the draining lymph nodes.^58^ In this case, blocking exosomal PD‐L1 may serve as an effective strategy inhibit tumor growth. Moreover, exosomes derived from cancer‐associated lymphatic ECs hinder cytotoxic T cell responses and facilitate immune evasion by inhibiting HMBOX1‐SOCS8 expression and activating JAK1/STAT1 signaling. This, in turn, enhances lymphatic PD‐L1 expression and lymphangiogenesis, while impairing CD3^+^ T cell immunity.^116^ Additionally, it has been demonstrated that exosomes derived from non‐small cell lung cancer (NSCLC) mediate resistance to anti‐PD1 therapy through the induction of CD8^+^ T‐cell depletion, highlighting a potential therapeutic target for treating NSCLC patients.^117^ Mechanistically, circUSP7, identified in the plasma of NSCLC patients, is primarily secreted by NSCLC‐derived exosome. It inhibits the secretion of IFN‐γ, TNF‐α, granzyme‐B, and perforin from CD8^+^ T cells. CircUSP7 impairs CD8^+^ T cell functionality by upregulating the expression of Src homology region 2‐containing protein tyrosine phosphatase 2 via miR‐934 sponging. Moreover, exosomes derived from BC have been shown to accumulate in the lungs and decrease T cell and NK cell populations.^118^ Specifically, the continuous accumulation and uptake of EO771‐derived exosomes in the lung were found to recruit CD11b^+^/Ly6Cmed granulocytic MDSCs (gMDSCs), while simultaneously reducing the frequency of T cells and NK cells, thus establishing an immunosuppressive microenvironment. gMDSCs, a subpopulation of immature myeloid cells, expand during cancer development and are associated with poor prognosis, primarily due to their ability to suppress T cell‐mediated immunity.^118^ More importantly, MDSCs, which play a crucial role in immune evasion during cancer progression, are a heterogeneous population of immature myeloid cells. MDSCs suppress the immune response by inducing Tregs and promoting the differentiation of macrophages into the M2 phenotype.^119^, ^120^, ^121^ Importantly, exosomes derived from MDSCs can directly accelerate tumor cell proliferation and metastasis by delivering miR‐126a. In addition, TEXs promote the development and immunosuppressive functions of MDSCs by upregulating the expression of antiapoptotic proteins and activating the STAT1/3 pathway.^121^ Furthermore, TEXs promote the production of inhibitory molecules in MDSCs, thereby enhancing their immunosuppressive activity within the TME. These findings emphasize the importance of TEXs within the tumor immunity of MDSCs. Briefly, TEXs are actively involved in the interaction between various immune and nonimmune cells within the TME, thereby influencing both cancer progression and immune escape. These interactions have substantial implications for tumor's response to, and resistance against, immunotherapy. Consequently, a deeper understanding of the exosome‐mediated signaling network within the TME, and its role in cancer‐induced immunosuppression, is crucial for developing more effective therapeutic strategies.

## 4 THE IMMUNE STIMULATORY EFFECTS OF EXOSOMES

Interestingly, alongside their immunosuppressive effects on cancer, exosomes can also exert immunostimulatory effects. This dual role stems from their ability to mirror the functions of their parental cells. Consequently, exosomes derived from immune cells naturally possess the capacity to mediate and enhance immune responses (Table S2). When stimulated by tumor cells, immune cells become activated, initiating the elimination of cancer cells. For example, cytotoxic T lymphocytes (CTL), once activated, secrete granules of perforin (a membrane destroying protein), granulysin, and granzyme, which work together to destroy cancer cells. Immune cell‐derived exosomes contain similar perforin‐like granules, enabling them to enter the cytoplasm of target cells and trigger the activation of cell death pathways.^122^ Importantly, immune cell‐derived exosomes play an important role in facilitating communication between innate and adaptive immune cells, as well as bridging the gap between the immune response and tumor cells. Different types of innate immune cells secrete and uptake exosomes, which allow them to interact with adaptive immune cells, including DCs, T cells, and B cells. Furthermore, TEXs can present neoantigens or directly activate NK cells or macrophages, thereby activating cellular immune responses. For example, TEXs transfer tumor antigen such as heat shock protein (HSP70–80) and major histocompatibility complex (MHC)‐I molecules to DCs, inducing CD8^+^ T cell‐dependent antitumorigenic effects.^62^ Additionally, exosomes can activate immunity by reprogramming TAMs into M1‐like macrophages, presenting antigens, and mediating cytotoxic effects mediated by NK‐derived exosomes, ultimately inhibiting tumor progression.

### 4.1 Reprogramming tumor associated macrophages into M1‐like macrophages

Macrophages play a prominent role in regulating innate and adaptive immune responses, contributing to pathogen clearance and antitumor immunity. During their activation, macrophages are influenced by IRF/STAT signaling pathways, which drive their differentiation into inflammatory (M1) or regulatory (M2) subtypes in response to a variety of cytokine stimuli. M1 macrophages, the predominant phenotype in normal immune responses, are essential for antitumor immunity, as they promote inflammation, phagocytosis, and activate cytotoxic T cells. In contrast, M2 macrophages exhibit protumorigenic activity by producing immunosuppressive cytokines, including IL‐4, IL‐10, and TGF‐β. Within the TME, factors like hypoxia, nutrient deprivation, excess lactate, and immunosuppressive cytokines drive the polarization of tumor‐infiltrating macrophages into TAMs with an M2‐like phenotype.

**FIGURE 6:**
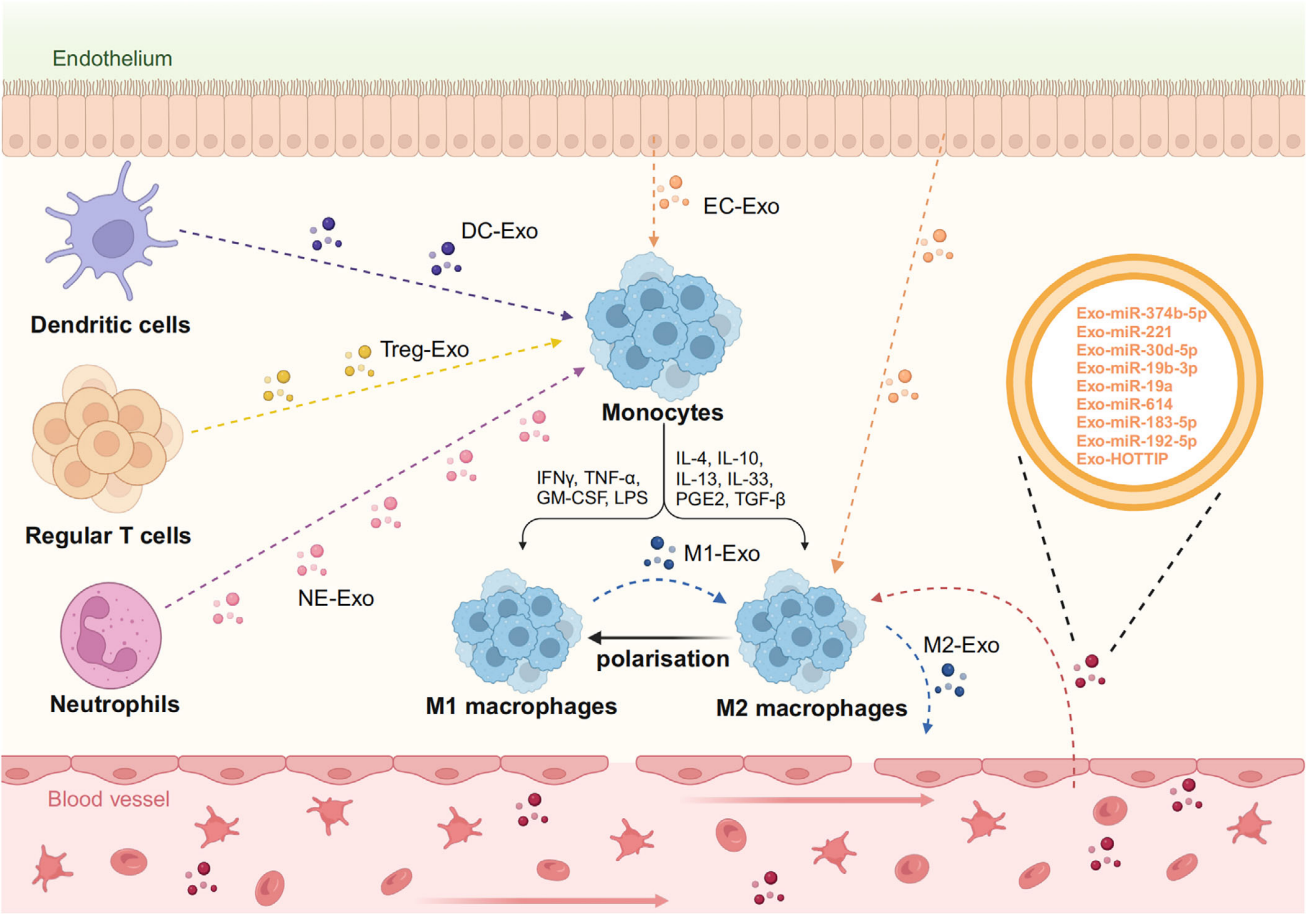
Exosomes are involved in the regulation of macrophage polarization. M2 macrophage polarization occurs when M0 macrophages are exposed to specific stimuli, including cytokines like IL‐4, IL‐13, IL‐10, and TGF‐β secreted byTh2. M1 macrophage polarization occurs when M0 macrophages are exposed to specific stimuli, including cytokines like IFNγ, GM‐CSF, and TGF‐α secreted byTh1 (created in BioRender.com).

Recent studies have highlighted the crucial role of macrophage‐derived exosomes in macrophage polarization^123^ (Figure 6). Notably, exosomes derived from M1 macrophages, along with their key molecule lncRNA HOTTIP (distal HOXA transcript), have been shown to inhibit the progression of head and neck squamous cell carcinoma (HNSCC). Specifically, M1‐derived exosomes and HOTTIP upregulate the TLR5/NF‐κB signaling pathway by competitively sponging miR‐19a‐3p and miR‐19b‐3p, which polarizes TAMs to an antitumor M1 phenotype. This activation of the innate immune system ultimately inhibited the progression of HNSCC.^124^ In addition, a growing number of studies have confirmed that miRs carried in exosomes can be transferred to TAMs, thereby affecting their polarization.^125^, ^126^ For example, exosomes derived from human BC, carrying miR‐33, have been shown to induce polarization of TAMs toward the M1 macrophage phenotype.^127^ Specifically, miR‐33 acts as a metabolic regulator by targeting the MAPK pathway, reducing fatty acid oxidation, and promoting M1 macrophage polarization. Furthermore, exosomal miR‐130 secreted by BC cells targets TAMs and participates in the PTEN/PI3K/AKT pathway, which, in turn, promotes TAM polarization into the M1 macrophage phenotype.^128^ Since M1 macrophages function as tumor suppressors in cancers, reprogramming TAMs into M1‐polarized macrophages may be an effective strategy for cancer treatment. For example, NF‐κB p50 inhibits the proinflammatory responses of M1 macrophages in TAMs, while miR‐511‐3p downregulates the genes involved in the protumoral activity of TAMs and inhibits tumor growth. Therefore, NF‐κB p50 siRNA and miR‐511‐3p were loaded into M1‐derived exosomes to enhance their M1 polarization activity.^125^ Recently, researchers have focused on converting TAMs into M1 macrophages with the antitumor phenotype.^129^, ^130^ For example, Zhen et al.^129^ developed P‐I@M1E/AALs by integrating M1‐like macrophage‐derived Exos with AS1411 aptamer‐conjugated liposomes. This approach was aimed at increasing the population of proinflammatory M1‐like macrophages in the mouse model of TNBC. Thus, understanding and harnessing the potential of exosomes to regulate macrophage polarization toward the M1 phenotype holds promise for enhancing the effectiveness of cancer immunotherapy.

### 4.2 Antigen presentation

**FIGURE 7:**
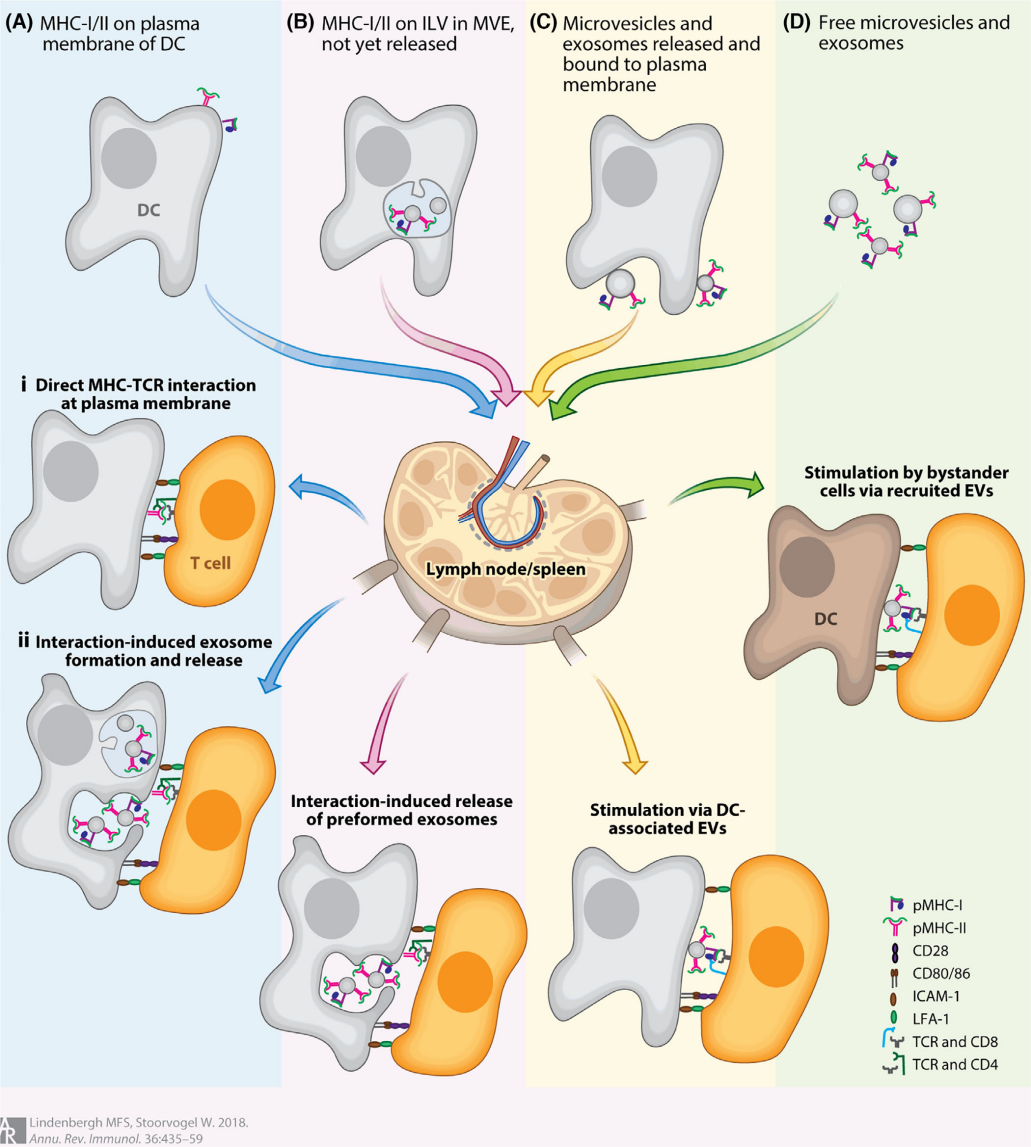
Interaction of DEXs with T cells. (a) Peripheral DCs present peptide‐MHC complexes at their plasma membrane. (b) Peripheral DC packages peptide‐MHC complexes into multivesicular endosomes (MVE), and DC interacts with target cells and releases their preformed intraluminal vesicles (ILV) as exosomes. (c) DC‐released exosomes and/or microvesicles can directly present their peptide‐MHC complexes to T cells. (d) DC‐released free microvesicles or exosomes flow to the lymph nodes or spleen. They can activate T cells either directly or after being recruited by bystander APCs (reprinted with permission from Ref.^138^).

Antigen‐presenting cells (APCs), which include DCs, macrophages, and B lymphocytes, are of vital importance in both innate and adaptive immune responses.^131^ Among these, DCs are considered the most potent APCs due to their ability to efficiently recognize and present invading antigens or aberrant self‐antigens. DCs themselves are highly diverse, with multiple subtypes that specialize in different immunological functions. In the context of antitumor immunity, exosomes derived from DCs (DEXs) have been shown to play an important role in antigen presentation.^132^ A previous study has shown that exosomes derived from α‐fetoprotein‐expressing DCs can trigger potent antigen‐specific antitumor immune responses and reshape the TME. These DEXs led to a notable increase in CD8^+^ CTL, as well as a significantly higher CD8^+^/CD4^+^ T‐cell ratio compared with controls in hepatocellular carcinoma mice. As a result, tumor growth was significantly inhibited, and survival was prolonged.^133^ Exosomes play a key role in transporting and presenting functional MHC–peptide complexes, which regulate the activation of antigen‐specific T cells through both direct and cross‐presentation pathways.^134^, ^135^ The molecular composition of DEXs comprises the surface expression of functional MHC‐I and MHC‐II molecules, which can activate CD8^+^ and CD4^+^ T cells, respectively. Additionally, DEXs contain costimulatory molecules and other components that further enhance T cell activation (Figure 7). Components on the surface membranes of DEXs, such as integrins and ICAM, facilitate their binding and uptake by APCs for indirect antigen presentation.^42^ Once internalized by APCs, DEXs peptide/MHC complexes can be reprocessed within the APCs, transferring antigenic peptides from DEXs' MHCs to the MHCs of the APCs. This process of indirect antigen presentation can effectively stimulate T cell responses.^136^, ^137^

In addition, DEXs carry several functional molecular substances on their surface, which may play key roles in antigen presentation. For example, CD86 is a costimulatory molecule that is critical for enhancing T cell activation during the antigen presentation process.^139^, ^140^ Members of the Hsp70 family are other components of DEXs that occur in the DC endocytic compartment. They are essential for immunogenicity due to their roles as antigenic chaperones and their involvement in MHC loading.^141^ Notably, rather than being internalized, DEXs are incorporated into the surface membrane of APCs by binding directly to them. This facilitates the immediate recognition of MHC‐presented peptides by T cells without requiring further antigen processing.^142^, ^143^ In the context of MHC class I molecules, activated CD8^+^ T cells play a central role in eliminating tumor cells through direct interactions with tumor antigenic peptides.^144^ In addition, B‐lymphocyte‐derived exosomes carry MHC‐II molecules that present peptides to CD4^+^ T cells, thereby eliciting antigen‐specific responses.^145^ Furthermore, macrophage‐derived exosomes can also initiate CD8^+^ T cell responses by cross‐presenting antigens associated with dead cells, similar to the way macrophages themselves do.^146^ In summary, APC‐derived exosomes are equipped with costimulatory molecules and relevant tumor‐specific antigens loaded onto MHC‐I and MHC‐II, highlighting their significant potential in cancer immunotherapy.

### 4.3 Cytotoxic effects mediated by NK‐derived exosomes

NK cells, essential members of antibody‐dependent cell‐mediated cytotoxicity, possess an intrinsic ability to recognize and eliminate virus‐infected and tumor cells. Owing to their capacity to modulate the immune response via cytokines, NK cells play a vital role in anticancer immunity. Their cytotoxic activity has been identified as a critical mechanism in NK cell‐based immunotherapy. Notably, NK cell‐derived exosomes, endowed with cytotoxic activity against tumor cells, can promote antitumor therapies through interactions with the tumor and/or immune cells. For example, miR‐186, a tumor suppressor derived from NK cell exosomes, exhibits cytotoxic effects against MYCN‐amplified neuroblastoma.^147^ In addition, TGF‐β in the TME can stimulate the STAT3 and SMAD2/3 pathways in NK cells, which suppress NK cell cytotoxicity and, consequently, inhibit their innate antitumor activity. When miR‐186, carried in NK‐cell‐derived exosomes, is overexpressed, TGF‐β receptors are downregulated, leading to the inhibition of neuroblastoma cell growth and counteracting the TGF‐β‐dependent immune escape mechanism. In addition to exosomal RNA, exosomal killer proteins from NK cells, such as Fas ligands and perforin molecules, also exert cytotoxic activity against various human tumor target cells and activated immune cells.^148^ Furthermore, NK cells exhibit rapid immunity against metastatic or hematological malignancies, and NK cell‐derived exosomes also demonstrate cytotoxic effects on melanoma cells.^149^ This is primarily because NK cell‐derived exosomes secrete TNF‐α, which affects cell proliferation signaling pathways.^149^ Understanding the regulatory mechanisms of NK‐derived exosomes can provide new insights into the modulation of immune responses and offer potential therapeutic targets for diseases associated with NK cell dysfunction. Recently, a study showed that chimeric antigen receptor‐NK (CAR‐NK) cell‐derived exosomes contain the surface membrane molecules of CAR‐NK cells, ensuring a lethal strike against target cells. Additionally, these exosomes exhibit excellent tumor‐targeting potential due to the presence of an antigen‐recognition region in the form of a single‐chain variable fragment (scFv).^150^ Leveraging the unique features of CAR‐NK cell‐derived exosomes, Tao et al. ^151^ designed a nanoplatform that combining CAR‐NK cell‐derived exosomes with a nanobomb. This innovative nanoplatform integrates HER2‐targeted brain metastasis anchoring capabilities for BC and an on‐demand release mechanism to enhance ferroptosis in tumor therapy. In this context, NK‐derived exosomes serve both as carriers and as agents that exert their own cytotoxic effects.

## 5 CLINICAL APPLICATION OF EXOSOMES

Notably, exosomes possess multiple advantages as potential therapeutic targets owing to their homing characteristics and ability to regulate tumor progression. While numerous in vitro studies have explored targeting exosomes for cancer therapy across various research domains, only a limited number of clinical trials have been conducted. Strategies such as inhibiting the production of immunosuppressive exosomes or leveraging exosomes with immune‐activating properties to deliver therapeutic agents have proven effective in impeding tumor progression. Targeting both exosomes and cancer cells plays an important role in inhibiting cancer progression and may serve as a promising therapeutic strategy for future tumor treatment. Additionally, exosome‐mediated molecular transport has become increasingly recognized for its critical role in various diseases, such as cancer and inflammatory diseases. For example, TEXs and exosomes derived from immune cells can activate the immune response by transferring antigens to APCs. This process activates both CD4^+^ T and CD8^+^ T cells, enhancing the antitumor response and inhibiting tumor progression.^152^ Recently, exosomes have been used as “natural nanoparticles” as drug delivery vehicles because of their low cytotoxicity and excellent targeting and homing specificity. Exosomes can be engineered to load exogenous drugs, such as nucleotides and proteins, and subsequently transport them to recipient cells. More importantly, exosome‐based drug delivery systems have the advantages of high biocompatibility, low immunogenicity, innate cell targeting ability, and ease of chemical and genetic manipulation. Therefore, exosomes can be used as an integrated platform for delivering multiple drugs or therapeutic strategies, enabling the synergistic activation of various stages in the tumor immune cycle and thereby enhancing antitumor immune responses. Several ongoing clinical trials are exploring the use of exosomes in cancer immunotherapy, focusing on diseases such as chronic kidney disease, NSCLC (ClinicalTrials.gov identifier: NCT05854030), and non‐Hodgkin B‐cell lymphomas (ClinicalTrials.gov identifier: NCT03985696; NCT01159288). These trials aim to evaluate the safety, efficacy, and potential benefits of exosome‐based treatments. Although exosome immunotherapy has made some progress in clinical research, it remains in the early stages of clinical research. Below, we will explore two facets of exosomes: their role as biomarkers for immunotherapy efficacy and cancer detection, and their function as biological delivery vehicles.

### 5.1 Exosomes as biomarkers of immune activation in immunotherapy

Cancer is a complex and heterogeneous disease with dynamic characteristics.^117^ Therefore, the detection of cancer biomarkers is essential for determining cancer status. Currently, liquid tumor biopsies primarily rely on blood‐based markers to provide specific information about tumors.^153^, ^154^, ^155^ These markers mainly include circulating tumor cells, circulating tumor DNA, and exosomes. Body fluids contain exosomes with unique biogenesis, and the cargo within these exosomes, including nucleic acids, lipids, and proteins, plays a crucial role in detecting cancer biomarkers. Compared with circulating tumor cells and circulating tumor DNA, exosomes possess higher stability and abundance, making them valuable for cancer detection.^156^, ^157^, ^158^, ^159^ Monitoring the contents of exosomes, such as specific proteins or miRs, can provide valuable insights into the effectiveness of immunotherapy. When the immune system is activated to combat cancer cells, exosomes carry distinct molecule cargo that can reflect the ongoing immune response. The detection of exosomal components in biological fluids, such as blood and urine, enables the identification of immune activation or suppression states. This approach holds significant potential for the early diagnosis of cancers, providing a new direction for both immunology research and clinical application.^160^ For example, an experiment involving patients with advanced NSCLC treated with PD‐1/PD‐L1 inhibitors revealed that the presence of exosomal hsa‐miR‐320d, hsa‐miR‐320c, and hsa‐miR‐320b could serve as potential indicators of improved efficacy in PD‐1/PD‐L1 immunotherapy.^161^ In addition, exosomal PD‐L1 has been identified as a potential early marker of adaptive immune activation following immunotherapy with PD‐1‐blocking antibodies in melanoma patients.^162^ While research on exosomes as indicators of immunotherapy efficacy is still in its early stages, several clinical studies are currently underway. One such study aims to develop and assess the potential of circulating exosomes as companion diagnostic biomarkers to predict responses to immunotherapy in patients with renal cell carcinoma (ClinicalTrials.gov identifier: NCT05705583). Moreover, an observational, prospective, two‐center study involving 50 patients with advanced squamous cell carcinoma undergoing surgical intervention is being conducted. This study aims to investigate the efficacy of serum exosomal miRNA as a biomarker for predicting the therapeutic effect of immunotherapy combined with chemotherapy (ClinicalTrials.gov identifier: NCT05854030).

In recent years, exosome‐based liquid biopsy strategies have attracted much attention in clinical applications, with several methods and commercial products developed for cancer diagnosis. For example, Zeng et al.^163^ developed a robust and ultrasensitive method for detecting TEXs by targeting exosomal mucin 1 and PD‐L1. This method integrates aptamers, DNA nanomachines, rolling circular amplification, and label‐free, homogeneous electrochemical techniques.^163^ The core of this separation‐free and label‐free approach relies on the specific interactions between methylene blue and doxorubicin with G‐quadruplex structures and DNA nanospheres, respectively. The employed strategy facilitated accurate discrimination between lung cancer patients (*n* = 25) and healthy donors (*n* = 12), yielding a specificity of 100% (12 out of 12), sensitivity of 92% (23 out of 25), and an overall accuracy of 94.6% (35 out of 37). Moreover, it achieved an impressive area under the receiver operating characteristic curve value of 0.97. Furthermore, the exosomics company developed a peptide‐based affinity isolation kit to isolate plasma exosomes from patients with metastatic melanoma, followed by the detection of BRAF^V600E^ mutant DNA using digital PCR. Compared with ctDNA‐based liquid biopsy, exosomes isolated via the polymer‐assisted method demonstrated a higher prevalence of BRAF^V600E^ mutations.^164^

Exosome biomarkers not only reflect the efficacy of immunotherapy but also offer insights into abnormal cancer signaling, tumor stromal responses, and the physiological state of secretory cells, all of which can reflect the host's response to cancer pathology. To date, exosomes have been extensively used as biomarkers for early cancer diagnosis in numerous clinical trials (Table S3). As an illustration of cutting‐edge research, within the context of an ongoing clinical trial (ClinicalTrials.gov identifier: NCT04529915), researchers systematically gathered exosomes from plasma samples of 420 patients with lung cancer and 150 healthy controls. The heterogeneity of exosomes was analyzed to distinguish between the healthy controls and lung cancer patients. Furthermore, biomarkers have been further examined in patients at different stages of lung cancer to enhance early detection and improve survival rates. Although the use of exosomes as a source of biomarkers presents challenges, such as issues with extraction and purification, they still hold a significant position in liquid biopsies for cancer.^165^ Collectively, exosomes are poised to revolutionize cancer progression monitoring and treatment efficacy assessment. Their ability to provide real‐time, noninvasive insights into tumor dynamics and therapeutic responses marks a significant advancement in personalized patient care and improved clinical outcomes.

### 5.2 Exosomes as carriers for immunotherapy

Exosomes, as drug carriers, possess the advantages of minimal toxicity, excellent biocompatibility, and inherent cell targeting capabilities, thereby augmenting the efficacy of cancer immunotherapy. Recently, with the introduction of engineering technologies, natural exosomes have been modified to develop exosome‐based drug delivery systems that play a pivotal therapeutic role in tumor immunotherapy (Table S4). For example, in preclinical studies, pH‐responsive exosome‐nanocouples have been developed by modifying the surface of M1 macrophage‐secreted exosomes with anti‐CD47 and anti‐SIRPα antibodies through acid‐cleavable of dibenzoylethylene bonds, targeting tumor cells. Upon selective cleavage in the acidic TME, these exosome‐nanocouples release the antibodies, blocking the interaction of SIRPα with CD47, thereby enhancing macrophage phagocytosis. In this study, all mice remained active throughout the observation period, and no lung metastases were detected. Immunofluorescence staining results indicated a significant increase in M1 macrophages and a notable decrease in M2 macrophages following pH‐responsive exosome‐nanocouples treatment. Quantitative analysis of the M1 marker (iNOS) and the M2 marker (arginase‐1) in tumor sections further confirmed an increased M1‐to‐M2 ratio. Consequently, the expression of Ki67, a key indicator of cell proliferation, was significantly suppressed.^166^ Similarly, the “don't eat me” mechanism of CD47 has been applied to exosome‐based immunotherapy. Koh et al.^167^ developed an exosome‐based antagonist featuring SIRPα variants on the exosome surface and then investigated the efficacy of systemically administered SIRPα‐exosomes in HT29 tumor‐bearing BALB/c nude mice. Their findings indicate that a CD47 blocking strategy utilizing SIRPα‐exosomes can effectively counteract CD47‐mediated inhibitory signaling. Furthermore, the potential side effect of anti‐CD47 monoclonal antibody treatment can be avoided, as only a minimal amount of exosomal antagonists is required to induce tumor regression in vivo.^168^ Additionally, as a new drug delivery system, exosomes have emerged as potent candidates in cancer immunotherapy, offering the potential for groundbreaking antitumor vaccines. This is due to their ability to transport antigens and facilitate antigen presentation to APCs, including DCs and macrophages.^169^ These exosomes can significantly enhance T cell activation, thereby fostering a robust adaptive immune response. Due to the surface membrane components of DEXs that interact with other immune cells, they have the potential to be modified into cell‐free antitumor vaccines, offering a novel approach to antitumor immunotherapy.^170^ Vaccination with tumor antigen‐loaded DEXs has been developed as an immunotherapeutic approach.^171^, ^172^ In a vaccination trial using tumor antigen (MART‐1)‐loaded DEXs, a phase I trial demonstrated the safety and feasibility of the DEXs vaccine. However, as this immunotherapy was unable to monitor the induction of T cells in patients, a second generation of DEX was developed with improved immunostimulatory capabilities. Maintenance immunotherapy was administered to 47 patients with advanced unresectable NSCLC who exhibited a response or stability following induction chemotherapy with DEX‐based treatment, aiming to enhance the progression‐free survival rate at 4 months in this patient cohort (ClinicalTrials.gov Identifier: NCT01159288). In addition to using immune cell‐derived exosomes as vaccines, there have also been studies on the preclinical application of TEXs for vaccination purposes. Despite their immunosuppressive effects, TEXs are also effective antigen delivery vehicles. TEXs can carry tumor‐specific antigens, providing effective signals to the immune system to activate an immune response against the tumor. For instance, immunogenic cell death‐inducible factors such as human neutrophil elastase and Hiltonol were encapsulated in BC‐derived exosomes, which were developed as an in situ vaccine. This vaccine exhibited potent antitumor activity in mouse models and BC‐like organs by promoting in situ activation of cDC1s, thereby improving subsequent tumor‐responsive CD8^+^ T cell responses.^173^ However, as mentioned earlier, in addition to tumor antigens, TEXs also carry several oncogenes, mRNAs, and miRs that induce tumor progression and metastasis. Therefore, the safety of TEXs‐based vaccines remains uncertain. Interestingly, researchers are constantly exploring ways to harness the properties of TEXs to design new immunotherapy strategies, aiming to combine their immunosuppressive effects with their potential as antigen delivery carriers to develop more effective treatment options.

The progress of exosome‐based clinical trials in immunotherapy is relatively slow, primarily constrained by technological challenges, regulatory hurdles, and funding limitations. Future research on exosomes as carriers for immunotherapy will concentrate on enhancing the efficiency of targeted delivery and assessing their roles in various immune microenvironments. Additionally, investigating the use of exosomes in combination with other therapeutic agents, such as immune checkpoint inhibitors, will constitute a pivotal strategy to augment therapeutic efficacy. At the same time, strengthening clinical validation of the safety and efficacy of exosomes will establish a robust foundation for their widespread application in cancer immunotherapy.

## 6 CONCLUSION AND FUTURE PERSPECTIVES

Over the past decades, exosomes have been the subject of considerable research not only in basic science but also in drug delivery, particularly in cancer immunotherapy. However, the heterogeneity of exosomes and their diverse origins has sparked debate regarding their role in immunotherapy. Most TEXs exert immunosuppressive effects by preparing a premetastatic niche, promoting angiogenesis, remodeling stromal cells, reprogramming metabolism, and facilitating immune escape. In contrast, some exosomes of immune cell origin have stimulatory effects that can inhibit tumor progression by reprogramming TAMs into M1‐like macrophages, remodeling the TME, presenting antigens, and exerting cytotoxic effects. Many of these differences stem from diverse functions of exosomes, including intercellular communication and immune regulation. In this review, we elucidate the mechanisms by which exosomes contribute to immunosuppression and immune activation, as well as their clinical application in immunotherapy. Beyond their promising role in cancer immunotherapy, exosomes also exhibit significant potential in stem cell therapy^174^, ^175^, ^176^ and gene therapy.^177^, ^178^ Exosomes secreted by stem cells can transport a wide array of bioactive molecules, facilitate tissue regeneration, modulate repair and inflammation processes, and enhance the efficacy of stem cell therapies.^179^, ^180^, ^181^ Based on these properties, the application of exosomes in stem cell therapy offers a new strategy for regenerative medicine, immune regulation and targeted therapy. Similar to their use as vectors for immunotherapy, exosomes can also be used as vectors for gene therapy. As gene delivery vehicles, exosomes can effectively transport siRNA, mRNA, and gene editing tools, enhance targeting, and reduce immune response.^182^ Exosomes have shown broad potential for clinical applications across various fields, including cancer, stem cell therapy, cardiovascular diseases,^183^ and nervous system diseases.^184^ They are expected to drive further advancements in clinical applications and development in the future.

In the field of immunotherapy, exosomes show several advantages over conventional chemotherapy. Although engineering has led to enhanced therapeutic effects, there are still few well‐defined standards, exosome‐based clinical trials have achieved limited success. Owing to the biological complexity of exosomes, a deeper understanding of the cargo and membrane surface molecules they carry is necessary to better address the controversial outcomes of exosome‐based immunotherapy. Further studies are required to investigate the role of exosomes in cancer progression through intercellular communication, which is important for advancing cancer therapy. Undoubtedly, exosome‐based immune therapies are on the brink of clinical translation; however, they still face significant translational challenges. The main challenges in applying exosomes to cancer treatment include, but are not limited to: (1) avoiding immune responses produced by nonautologous exosomes. The use of exosomes as a therapeutic tool may raise safety concerns, such as over‐activation of the immune system or adverse reactions. Therefore, researchers must conduct rigorous safety assessments to ensure the safety of exosome‐based therapies; (2) the need for extensive systematic studies in vivo to demonstrate the efficacy of exosomes in immunotherapy; (3) improving the tumor‐killing effect of T cells when utilizing exosomes as vaccines; (4) efficiently loading exogenous active molecules (e.g., proteins, nucleic acids, and lipids) into exosomes and further enhancing target delivery efficiency, and (5) ensuring quality control of exosomes administered to patients while addressing technical challenges associated with clinical‐grade production. Due to the complexity of their biology, exosomes act as double‐edged swords in immunotherapy. Therefore, it is crucial to establish quality standards for exosomes and improve their in vivo efficacy before translating them from the bench to the bedside.

In this review, we aim to elucidate the current mechanisms by which exosomes contribute to immunotherapy. However, there are notable limitations in understanding the role of exosomes within the TME. First, the origin and composition of exosomes are inherently complex, making their accurate isolation and analysis both in vivo and in vitro challenging. Second, the heterogeneity of exosomes significantly impacts their efficacy in immunotherapy, adding to the complexity and challenges of research. Third, while some functions of exosomes have been identified, their complete mechanisms—particularly their specific actions within the TME—remain inadequately understood and warrant further investigation. Additionally, challenges remain regarding the stability and reliability of exosomal biomarkers for clinical applications. Therefore, future advancements in this field should prioritize ongoing research into the mechanisms of tumor immunity and exosome interactions within the TME, which are crucial for optimizing innovative immunotherapies. Furthermore, advanced bioengineering techniques should be employed to enhance the targeting capabilities and therapeutic payload delivery of exosomes. Overall, research on exosomes in immunotherapy will concentrate on a comprehensive analysis of molecular mechanisms, innovative applications in oncology and autoimmune disorders, and the development of personalized treatment protocols. These efforts will not only enhance therapeutic outcomes but also offer patients with a broader array of options and novel treatment pathways. With sustained investment in scientific research and technological innovation, we are confident that the significance of exosomes in immunotherapy will continue to grow, ultimately leading to more precise and effective treatment strategies for cancer patients.

## AUTHOR CONTRIBUTIONS

Zhaogang Yang, Jiayi Chen, and Simiao Wang were responsible for conceptualization and designing of this review. Jiayi Chen and Siyuan Hu drafted and revised the manuscript. Jiayi Liu and Hao Jiang participated in the design of the figures and revision of the manuscript. All authors have read and approved the final version of the manuscript.

## CONFLICT OF INTEREST STATEMENT

The authors declare no conflicts of interest.

## ETHICS STATEMENT

Not applicable.

## Supporting information



Supporting Information

## Data Availability

Data availability is not applicable to this review as no new data were created or analyzed in this study.
