# Supplementary material for: Exosomes: a double‐edged sword in cancer immunotherapy
Source: MedComm (2020). 2025 Feb 17;6(3):e70095. doi: 10.1002/mco2.70095 (PMC11831209; doi:10.1002/mco2.70095)
Supplement: Supplementary file 1 — Supporting Information [file MCO2-6-e70095-s001.docx]

**Exosomes: a double-edged sword in cancer immunotherapy**

Jiayi Chen^1^, Siyuan Hu^1^, Jiayi Liu^1^, Hao Jiang^1^, Simiao Wang^1,^*, Zhaogang Yang^1,^*

^1^ School of Life Sciences, Jilin University, Changchun, 130012 China

* Corresponding author. Tel.: +86-431-85155320; Fax: +86-431-85155320.

E-mail addresses: smwang23@mails.jlu.edu.cn (S. W.); [zhaogangyang@jlu.edu.cn](mailto:zhaogangyang@jlu.edu.cn) (Z.Y.)

**Table S1 Effect of exosomes from different sources in immune suppression**

| **Cancer Type** | **Source** | **Molecular** | **Mechanism** | **Effect** | **Reference** |
| --- | --- | --- | --- | --- | --- |
| Hepatocellular Carcinoma | Hepatocellular Carcinoma cells | circTMEM181 | Sponging miR-488-3p and upregulates CD39 expression in macrophages thereby activating the ATP-adenosine pathway to produce more adenosine. | Impairing CD8 T cell function which in turn promotes immunosuppression and drives anti-PD1 resistance. | ^1^ |
|  |  | circUHRF1 | Regulation of miR-449c-5p/TIM-3 pathway to inhibit NK cell function. | Impairing the sensitivity of hepatocellular carcinoma cells to anti-PD1 therapy. | ^2^ |
|  |  | miR-142-3p | Targeted regulation of SLC3A2 promotes HBV-induced iron death in M1 macrophages. | Promoting proliferation, migration and invasion of hepatocellular carcinoma cells. | ^3^ |
|  |  | miR-143-3p | Upregulation of the target gene MARCKS in tumor-associated macrophages. | Impacts M2 polarization and immune escape, resulting in poor tumor prognosis. | ^4^ |
|  |  | ZFPM2-AS1 | Direct targeting of MiRNA-18b-5p, sponging miR-18b-5p triggers PKM expression and regulates glycolysis through PKM in a HIF-1α-dependent manner. | Promotion of M2 polarization and macrophage recruitment enhances tumor growth, metastasis and M2 infiltration. | ^5^ |
|  |  | miR-223 | Macrophage regulation through down-regulation of PDCD4 inhibits inflammation. | Promotes angiogenesis which in turn supports tumor growth. | ^6^ |
|  | Tumor-associated macrophages | lncMMPA | Polarizes M2 macrophages, acts as a microRNA sponge that interacts with miR-548 and increases mRNA levels of ALDH1A3. | Promotes glucose metabolism and cell proliferation in HCC. | ^7^ |
|  | M2 macrophages | miR-27a-3p | Targeting and downregulating TXNIP expression in HCC cells. | Promotes cancer stemness, proliferation, drug resistance, migration, invasion and tumorigenicity. | ^8^ |
|  | Macrophages | miR-92a-2-5p | Targeting the 3' untranslated region (3'UTR) of AR mRNA to inhibit AR translation and regulate the PHLPP/p-AKT/β-catenin signaling pathway. | Suppression immune response and promoting tumor cell invasion. | ^9^ |
|  | Macrophages | IL-10 | Inhibition of macrophage and T-cell activity and inhibition of Th1 response promotes immune escape. | Suppression immune response and promoting tumor progression. | ^10^ |
| Melanoma | CD8+ T cells | uPAR | Promoting tumor cell invasion by modulating the extracellular protease system. | Correlates with expression of immune checkpoint receptors and may lead to immune evasion. | ^11^ |
|  |  | LFA-1 | Induction of dendritic cell apoptosis by down-regulation of pMHC I and through the Fas/FasL pathway. | Inhibits T cell activity, blocks CD8+ CTL response and suppresses tumor immune response. | ^12^ |
|  |  | FasL | Activation of ERK and NF-κB pathways increased MMP9 expression in tumors. | Induces T cell apoptosis and promotes immune escape and tumor metastasis. | ^13^ |
| Breast Cancer | Cancer-associated fibroblasts | miR-181d-5p | Targeting CDX2 and down-regulating the expression of CDX2 and HOXA5. | Promote tumor cell proliferation, invasion and migration, inhibit apoptosis. | ^14^ |
|  |  | miR-92 | Upregulation of PD-L1 and activation of the PD-L1 pathway. | Inhibits CD8+ T cell and NK cell function, enhances immune escape and promotes tumor immunosuppression. | ^15^ |
|  |  | miR-500a-5p | Down-regulates tumor suppressor USP28 and enhances tumor growth. | Inhibits USP28 expression and promotes tumor cell proliferation and metastasis. | ^16^ |
| Ovarian cancer | Ovarian Cancer cells | miR-155-5p | Targeted activation of NF-κB and JAK/STAT pathways. | Promoting tumor cell invasion. | ^17^ |
| Colorectal Cancer | Mesenchymal stem cells | TGF-β | Targeting the SMAD pathway to inhibit T cell and NK cell activity. | Promoting tumor cell migration, immune evasion. | ^18^ |
| Kidney Cancer | Mesenchymal stem cells | HGF | Activation of c-MET signaling pathway to promote tumor cell proliferation and metastasis. | Promoting tumor cell growth and immune evasion. | ^19^ |
| Neuroblastoma | CD8+ T cells | PD-1 | Inhibition of T cell activity through PD-1/PD-L1 pathway. | Inhibition tumor-specific CTL activity and promotes immune evasion. | ^20^ |
| Head and Neck Squamous Cell Carcinoma /Lung Cancer/Breast Cancer | Tumor | miR-21 | Down-regulating PTEN and activates PI3K/AKT pathway to inhibit T cell activation. | Enhancing immune escape and promotes tumor cell proliferation and immunosuppression. | ^21^ |

**Table S2 Effect of exosomes from different sources in immune activation**

| **Cancer Type** | **Source** | **Molecular** | **Mechanism** | **Effect** | **Reference** |
| --- | --- | --- | --- | --- | --- |
| Melanoma | CD4+ T cells | miR-25-3p, miR-155-5p, miR-215-5p, miR-375 | Activation of T cells through direct down-regulation of negatively regulated genes. | Promotes anti-tumor response of CD8+ T cells. | ^22^ |
|  | Melanoma cells | circPIK3R3 | Targeted down-regulation of miR-872-3p expression. | Increased CD8+ T cells activity and activation of tumor immunity. | ^23^ |
|  | Melanoma cells | ICAM-1  PD-L1 | Targeted up-regulation of LFA-1 expression. | Activation of T cells and tumor immunity. | ^24^ |
| Pancreatic | Natural Killer cells | IFN-γ | Activates immune response through JAK-STAT pathway. | Induction of apoptosis and immune activation in tumor cells. | ^25^ |
|  | Cancer-associated fibroblasts | Meflin | Inhibition of αSMA expression in CAFs and ECM remodeling (myofibroblast differentiation). | Associated with a subpopulation of anti-tumor CAFs, Meflin overexpression inhibits tumor growth. | ^26^ |
| Non-small Cell Lung Cancer | Cancer-associated fibroblasts | Versican (VCAN) | Inhibits tumor cell spread by modulating ECM structure. | Inhibits tumor growth and angiogenesis. | ^27^ |
|  | Non-small Cell Lung Cancer cells | miRNA-433 | Targeted down-regulation of the Wnt/β-catenin pathway. | Promotes CD4+ T cells and CD8+ T cells to infiltrate tumors | ^28^ |
| Lymphoma | CD4+ T cells | CD40L | Enhancing B-cell response and promotes B-cell activation, proliferation and antibody production. | Involving in the regulation of humoral immunity. | ^29^ |
| Endometrial Cancer | CD4+ T cells | miR-765 | Regulation of the miR765/PLP2 axis and consequent down-regulation of the PLP2-Notch signaling pathway. | Restriction of estrogen/ERβ-driven UCEC development. | ^30^ |
| Cervix | Tumor | Granzyme B and Perforin | Through perforation and activation of apoptotic pathways. | Direct tumor killing and induction of tumor cell apoptosis. | ^31^ |
| Hepatocellular Carcinoma | M1 Macrophages | miR-628-5p | Regulation of circFUT8/miR-552-3p/CHMP4B pathway and inhibition of m6A modification of circFUT8. | Down-regulation of human methyltransferase-like 14 (METTL14) expression inhibits hepatocellular carcinoma development. | ^32^ |
| Ovarian cancer | Ovarian Cancer cells | miR-155-5p | Targeted activation of NF-κB and JAK/STAT pathways. | Improves CD8+ T cells function and activate tumor immunity | ^17^ |
| Breast Cancer | Breast Cancer cells | PD-L1 | Targeted down-regulation of TGF-β expression. | Increased T cells activity and activation of tumor immunity. | ^33^ |
| Pancreatic and Colorectal Cancer | Tumor | HSP70 | Enhanced cytotoxicity through activation of NK cell migration and granzyme B release via the TLR4/NF-κB pathway. | Enhances immune response, promote apoptosis of tumor cells, inhibit tumor growth. | ^34^ |
| Head and Neck Squamous Cell Carcinoma /Lung Cancer/Breast Cancer | Tumor | miR-21 | Down-regulating PTEN and BRCC3 activates PI3K/AKT pathway. | Increased CD8+ T cells activity and activation of tumor immunity. | ^21^ |

**Table S3** **Exosomes as Cancer Biomarkers: Efficacy, Prediction, and Prognosis**

| **Disease** | **Source of Exosomes** | **Outcome Measures** | **ClinicalTrials.gov Identifier or Reference** |
| --- | --- | --- | --- |
| Lung Cancer | Blood Plasma | Evaluation of the distinction between healthy controls and lung cancer patients through analysis of exosomes | NCT04529915 |
| Early Lung Cancer | pathological specimens | exosomal micro-A in cancer tissue and para cancerous tissue | NCT03542253 |
| Prostate Cancer | Urinary, samples without DNA | Exosome gene expression signatures were associated with the presence or absence of high-grade prostate cancer on prostate biopsy | NCT02702856 |
| Breast Cancer | Blood Plasma | Positive predictive value of ctDNA/Exosome for breast cancer recurrence | NCT05955521 |
| Breast Neoplasms | Blood Plasma | characterizing protein surface markers and RNA profiles in tumor derived exosomes from breast cancer patients undergoing neoadjuvant chemotherapy | NCT01344109 |
| Lung Cancer | Blood | Evaluating size distribution, concentration and molecular profiling of pulmonary vein exosomes at inclusion | NCT04939324 |
| Colorectal Cancer | Blood | Diagnostic performances of markers derived from circulating tumor exosomes in the context of colorectal cancer, focusing on macromolecules, integrins, metallo proteases. | NCT04394572 |
| Lung Cancer | Serum Samples | The specific exosomal lncRNA generated by ELC can be used for non-invasive diagnosis of ELC with high sensitivity and stability. | NCT03830619 |
| Rectal Cancer | Serum Samples | Exosomal as Correlative Biomarker in Clinical Outcomes in Patients Undergoing Neoadjuvant Chemoradiation Therapy for Rectal Cancer | NCT03874559 |
| Thyroid Cancer | Urine Samples | Urinary exosomal thyroglobulin and galactin-3 prospectively predict the prognosis and recurrence of thyroid cancer | NCT03488134 |
| Gastric Cancer | Blood Sample | Liquid biopsy markers can be used as blood biomarkers for early detection and monitoring of early-onset gastric cancer | NCT06023121 |
| Thyroid Cancer | Urine Samples | AT exosomes may serve as biomarkers for the diagnosis and prognosis of malignant tumors in patients with thyroid cancer or undifferentiated thyroid cancer. | NCT02862470 |
| Melanoma | Peripheral Blood | The exosomes produced by senescent melanoma cells have an impact on melanoma development both in vitro and in vivo. | NCT02310451 |
| Prostate Cancer | Urine Samples | Potential exosomal microRNAs that could differentiate pathologically insignificant and significant PCa | NCT03911999 |
| Upper Gastrointestinal Tumors | Plasma Samples | Exosome specific protein biomarkers have been identified as indicative of early upper gastrointestinal tumors | NCT06278064 |
| Lung Squamous Carcinoma | Plasma Samples | Serum exosomal miRNA can predict the efficacy and prognosis of immunocombined chemotherapy in lung squamous cell carcinoma. | NCT05854030 |
| Lung Cancer | Serum Samples | The specific exosomal lncRNA generated by ELC can be used for non-invasive diagnosis of ELC with high sensitivity and stability. | NCT03830619 |
| Pancreatic Cancer | Blood Samples | The Exosome Protein Assay (ExoVerita^TM^) kit detects early pancreatic cancer with high sensitivity and specificity. | NCT05625529 |
| Breast Cancer | Plasma Samples | Detection of early biomarkers of exosomes and detection of breast cancer using SiMoA technology. | NCT05798338 |
| Breast Cancer | Plasma Samples | New SiMoA assay exosomes based on anti-CD63 and anti-CD9 antibodies to assess efficacy in neoadjuvant chemotherapy. | NCT05831397 |
| Small Cell Lung Cancer (SCLC) | Blood Sample | Exosomal long chain RNA (exLR) as a tumor biomarker reflects SCLC recurrence. | NCT05191849 |
| Pancreatic cancer | Blood Sample | Reduced GPC1 Expression Levels Correlate with Prolonged Patient Survival. | ^35^/  NCT03410030 |
| Melanoma | Blood Sample | An increase in the level of exosomal PD-L1, following and correlating positively with T cell reinvigoration, reflecting the presence of a successful anti-tumour immunity elicited by the anti-PD-1 therapy. | ^36^/  NCT02083484 |
| Hepatocellular carcinoma | Serum  Sample | Playing a role in the molecular mechanisms of VE-calmodulin and ZO-1 endothelial cell down-regulation, the miR-638 high-expression group possessed a lower rate of 2-y Disease Free Survival (DFS). | ^37^ |
| HER2-positive Breast cancer | Blood Sample | Expression levels of miR-1246 and miR-155 negatively correlated with Progression Free Survival (PFS) or Event Free Survival (EFS) in patients with early onset and metastasis. | ^38^ |
| Prostate cancer (PCa) | Blood Sample | Relative protein levels of caveolin-1 are associated with adverse clinical features and highly aggressive tumors in PCa. | ^39^ |

**Table S4 Advances in clinical studies of exosomes as delivery vehicles in cancer therapy**

| **Cancer Types** | **Treatment** | **Preclinical Study Results** | **Clinical Stage** | **Clinical Trial Results** | **ClinicalTrials.gov Identifier/Reference** |
| --- | --- | --- | --- | --- | --- |
| Pancreatic cancer | KrasG12D siRNA-loaded Mesenchymal stromal cell-derived exosomes | siRNA was delivered to effectively inhibit KRAS gene expression and reduce tumor growth. | Phase I | Preliminary demonstration of the safety of exosome therapy, with some patients in stable condition. | NCT03608631 |
| Non-Small Cell Lung Cancer | Tumor antigen-loaded dendritic cell-derived exosomes | Shown to be effective in activating immune responses in animal models. | Phase II | Good safety profile, enhances immune response, works on tumor cells. | NCT01159288 |
| Non-Small Cell Lung Cancer | Tumor antigen-loaded autologous dendritic cell-derived exosomes | Dexosomes have demonstrated significant antitumor activity in a mouse tumor model. | Phase I | Production of the DEX vaccine was feasible, treatment was well tolerated by patients, some patients experienced long-term stabilization of the disease and activation of immune effectors. | ^40^ |
| Metastatic melanoma | Autologous dendritic cell-derived exosomes pulsed with MAGE3 peptides | Exosomes pulsed with tumor peptides are more effective than peptides alone and are as effective as mature DCs in triggering MART1-specific CTLs and inhibiting tumor growth. | Phase I | The feasibility of large-scale exosome production and the safety of exosome drug delivery are emphasized. | ^41^ |
| Bladder Cancer | Chimeric Exosomal Tumor Vaccine Delivery System | In animal experiments, the vaccine significantly enhanced the anti-tumor immune response. | Phase I | Dose-escalation trials are under way to assess the immune effects and safety of the vaccine. | NCT05559177 |

# References

1. Lu JC, Zhang PF, Huang XY, et al. Amplification of spatially isolated adenosine pathway by tumor-macrophage interaction induces anti-PD1 resistance in hepatocellular carcinoma. *J Hematol Oncol*. Nov 27 2021;14(1):200.

2. Zhang PF, Gao C, Huang XY, et al. Cancer cell-derived exosomal circUHRF1 induces natural killer cell exhaustion and may cause resistance to anti-PD1 therapy in hepatocellular carcinoma. *Mol Cancer*. Jun 27 2020;19(1):110.

3. Hu Z, Yin Y, Jiang J, et al. Exosomal miR-142-3p secreted by hepatitis B virus (HBV)-hepatocellular carcinoma (HCC) cells promotes ferroptosis of M1-type macrophages through SLC3A2 and the mechanism of HCC progression. *J Gastrointest Oncol*. Apr 2022;13(2):754-767.

4. Ren X, Ju Y, Wang C, Wei R, Sun H, Zhang Q. MARCKS on Tumor-Associated Macrophages is Correlated with Immune Infiltrates and Poor Prognosis in Hepatocellular Carcinoma. *Cancer Invest*. Oct 2021;39(9):756-768.

5. Ji W, Bai J, Ke Y. Exosomal ZFPM2-AS1 contributes to tumorigenesis, metastasis, stemness, macrophage polarization, and infiltration in hepatocellular carcinoma through PKM mediated glycolysis. *Environ Toxicol*. Jun 2023;38(6):1332-1346.

6. Papadakos SP, Machairas N, Stergiou IE, et al. Unveiling the Yin-Yang Balance of M1 and M2 Macrophages in Hepatocellular Carcinoma: Role of Exosomes in Tumor Microenvironment and Immune Modulation. *Cells*. Aug 10 2023;12(16)

7. Xu M, Zhou C, Weng J, et al. Tumor associated macrophages-derived exosomes facilitate hepatocellular carcinoma malignance by transferring lncMMPA to tumor cells and activating glycolysis pathway. *J Exp Clin Cancer Res*. Aug 19 2022;41(1):253.

8. Li W, Xin X, Li X, Geng J, Sun Y. Exosomes secreted by M2 macrophages promote cancer stemness of hepatocellular carcinoma via the miR-27a-3p/TXNIP pathways. *Int Immunopharmacol*. Dec 2021;101(Pt A):107585.

9. Liu G, Ouyang X, Sun Y, et al. The miR-92a-2-5p in exosomes from macrophages increases liver cancer cells invasion via altering the AR/PHLPP/p-AKT/β-catenin signaling. *Cell Death Differ*. Dec 2020;27(12):3258-3272.

10. Zhou W, Yang F, Zhang X. Roles of M1 Macrophages and Their Extracellular Vesicles in Cancer Therapy. *Cells*. Aug 26 2024;13(17)

11. Del Rosso M, Margheri F, Serratì S, Chillà A, Laurenzana A, Fibbi G. The urokinase receptor system, a key regulator at the intersection between inflammation, immunity, and coagulation. *Curr Pharm Des*. 2011;17(19):1924-43.

12. Xie Y, Zhang H, Li W, et al. Dendritic cells recruit T cell exosomes via exosomal LFA-1 leading to inhibition of CD8+ CTL responses through downregulation of peptide/MHC class I and Fas ligand-mediated cytotoxicity. *J Immunol*. Nov 1 2010;185(9):5268-78.

13. Cai Z, Yang F, Yu L, et al. Activated T cell exosomes promote tumor invasion via Fas signaling pathway. *J Immunol*. Jun 15 2012;188(12):5954-61.

14. Wang H, Wei H, Wang J, Li L, Chen A, Li Z. MicroRNA-181d-5p-Containing Exosomes Derived from CAFs Promote EMT by Regulating CDX2/HOXA5 in Breast Cancer. *Mol Ther Nucleic Acids*. Mar 6 2020;19:654-667.

15. Li C, Teixeira AF, Zhu HJ, Ten Dijke P. Cancer associated-fibroblast-derived exosomes in cancer progression. *Mol Cancer*. Dec 1 2021;20(1):154.

16. Chen B, Sang Y, Song X, et al. Exosomal miR-500a-5p derived from cancer-associated fibroblasts promotes breast cancer cell proliferation and metastasis through targeting USP28. *Theranostics*. 2021;11(8):3932-3947.

17. Li X, Wang S, Mu W, et al. Reactive oxygen species reprogram macrophages to suppress antitumor immune response through the exosomal miR-155-5p/PD-L1 pathway. *J Exp Clin Cancer Res*. Jan 27 2022;41(1):41.

18. Lin Z, Wu Y, Xu Y, Li G, Li Z, Liu T. Mesenchymal stem cell-derived exosomes in cancer therapy resistance: recent advances and therapeutic potential. *Mol Cancer*. Sep 13 2022;21(1):179.

19. Weng Z, Zhang B, Wu C, et al. Therapeutic roles of mesenchymal stem cell-derived extracellular vesicles in cancer. *J Hematol Oncol*. Sep 3 2021;14(1):136.

20. Zhou Q, Wei S, Wang H, et al. T cell-derived exosomes in tumor immune modulation and immunotherapy. *Front Immunol*. 2023;14:1130033.

21. Cheng HY, Hsieh CH, Lin PH, et al. Snail-regulated exosomal microRNA-21 suppresses NLRP3 inflammasome activity to enhance cisplatin resistance. *J Immunother Cancer*. Aug 2022;10(8)

22. Shin S, Jung I, Jung D, et al. Novel antitumor therapeutic strategy using CD4(+) T cell-derived extracellular vesicles. *Biomaterials*. Oct 2022;289:121765.

23. Wang L, Shen K, Gao Z, et al. Melanoma Derived Exosomes Amplify Radiotherapy Induced Abscopal Effect via IRF7/I-IFN Axis in Macrophages. *Adv Sci (Weinh)*. Apr 2024;11(13):e2304991.

24. Zhang W, Zhong W, Wang B, et al. ICAM-1-mediated adhesion is a prerequisite for exosome-induced T cell suppression. *Dev Cell*. Feb 7 2022;57(3):329-343.e7.

25. Zhang M, Shao W, Yang T, et al. Conscription of Immune Cells by Light-Activatable Silencing NK-Derived Exosome (LASNEO) for Synergetic Tumor Eradication. *Adv Sci (Weinh)*. Aug 2022;9(22):e2201135.

26. Mizutani Y, Kobayashi H, Iida T, et al. Meflin-Positive Cancer-Associated Fibroblasts Inhibit Pancreatic Carcinogenesis. *Cancer Res*. Oct 15 2019;79(20):5367-5381.

27. Chang W, Zhu J, Yang D, et al. Plasma versican and plasma exosomal versican as potential diagnostic markers for non-small cell lung cancer. *Respir Res*. May 31 2023;24(1):140.

28. Liu B, Zhang R, Zhu Y, Hao R. Exosome-derived microRNA-433 inhibits tumorigenesis through incremental infiltration of CD4 and CD8 cells in non-small cell lung cancer. *Oncol Lett*. Aug 2021;22(2):607.

29. Lu J, Wu J, Xie F, et al. CD4(+) T Cell-Released Extracellular Vesicles Potentiate the Efficacy of the HBsAg Vaccine by Enhancing B Cell Responses. *Adv Sci (Weinh)*. Dec 2019;6(23):1802219.

30. Zhou WJ, Zhang J, Xie F, et al. CD45RO(-)CD8(+) T cell-derived exosomes restrict estrogen-driven endometrial cancer development via the ERβ/miR-765/PLP2/Notch axis. *Theranostics*. 2021;11(11):5330-5345.

31. Si C, Gao J, Ma X. Natural killer cell-derived exosome-based cancer therapy: from biological roles to clinical significance and implications. *Mol Cancer*. Jun 29 2024;23(1):134.

32. Wang L, Yi X, Xiao X, Zheng Q, Ma L, Li B. Exosomal miR-628-5p from M1 polarized macrophages hinders m6A modification of circFUT8 to suppress hepatocellular carcinoma progression. *Cell Mol Biol Lett*. Dec 6 2022;27(1):106.

33. Chatterjee S, Chatterjee A, Jana S, et al. Transforming growth factor beta orchestrates PD-L1 enrichment in tumor-derived exosomes and mediates CD8 T-cell dysfunction regulating early phosphorylation of TCR signalome in breast cancer. *Carcinogenesis*. Feb 11 2021;42(1):38-47.

34. Gastpar R, Gehrmann M, Bausero MA, et al. Heat shock protein 70 surface-positive tumor exosomes stimulate migratory and cytolytic activity of natural killer cells. *Cancer Res*. Jun 15 2005;65(12):5238-47.

35. Melo SA, Luecke LB, Kahlert C, et al. Glypican-1 identifies cancer exosomes and detects early pancreatic cancer. *Nature*. Jul 9 2015;523(7559):177-82.

36. Chen G, Huang AC, Zhang W, et al. Exosomal PD-L1 contributes to immunosuppression and is associated with anti-PD-1 response. *Nature*. Aug 2018;560(7718):382-386.

37. Yokota Y, Noda T, Okumura Y, et al. Serum exosomal miR-638 is a prognostic marker of HCC via downregulation of VE-cadherin and ZO-1 of endothelial cells. *Cancer Sci*. Mar 2021;112(3):1275-1288.

38. Zhang Z, Zhang L, Yu G, et al. Exosomal miR-1246 and miR-155 as predictive and prognostic biomarkers for trastuzumab-based therapy resistance in HER2-positive breast cancer. *Cancer Chemother Pharmacol*. Dec 2020;86(6):761-772.

39. Matijašević Joković S, Korać A, Kovačević S, et al. Exosomal Prostate-Specific Membrane Antigen (PSMA) and Caveolin-1 as Potential Biomarkers of Prostate Cancer-Evidence from Serbian Population. *Int J Mol Sci*. Mar 21 2024;25(6)

40. Morse MA, Garst J, Osada T, et al. A phase I study of dexosome immunotherapy in patients with advanced non-small cell lung cancer. *J Transl Med*. Feb 21 2005;3(1):9.

41. Escudier B, Dorval T, Chaput N, et al. Vaccination of metastatic melanoma patients with autologous dendritic cell (DC) derived-exosomes: results of thefirst phase I clinical trial. *J Transl Med*. Mar 2 2005;3(1):10.
